# The Temporal Dynamics of Differential Gene Expression in *Aspergillus fumigatus* Interacting with Human Immature Dendritic Cells *In Vitro*

**DOI:** 10.1371/journal.pone.0016016

**Published:** 2011-01-14

**Authors:** Charles O. Morton, John J. Varga, Anke Hornbach, Markus Mezger, Helga Sennefelder, Susanne Kneitz, Oliver Kurzai, Sven Krappmann, Hermann Einsele, William C. Nierman, Thomas R. Rogers, Juergen Loeffler

**Affiliations:** 1 Department of Clinical Microbiology, Trinity College Dublin, Dublin, Ireland; 2 J. Craig Venter Institute, Rockville, Maryland, United States of America; 3 Medizinische Klinik and Poliklinik II, Universitätsklinikum Würzburg, Würzburg, Germany; 4 Labor für Microarray Anwendungen, Interdisziplinäres Zentrum für Klinische Forschung, Würzburg, Germany; 5 Septomics Research Centre, Friedrich-Schiller-Universität Jena, Leibniz Institute for Natural Products Research and Infection Biology - Hans-Knöll-Institute, Jena, Germany; 6 Zentrum für Infektionsforschung, Universität Würzburg, Würzburg, Germany; Louisiana State University, United States of America

## Abstract

Dendritic cells (DC) are the most important antigen presenting cells and play a pivotal role in host immunity to infectious agents by acting as a bridge between the innate and adaptive immune systems. Monocyte-derived immature DCs (iDC) were infected with viable resting conidia of *Aspergillus fumigatus* (Af293) for 12 hours at an MOI of 5; cells were sampled every three hours. RNA was extracted from both organisms at each time point and hybridised to microarrays. iDC cell death increased at 6 h in the presence of *A. fumigatus* which coincided with fungal germ tube emergence; >80% of conidia were associated with iDC. Over the time course *A. fumigatus* differentially regulated 210 genes, FunCat analysis indicated significant up-regulation of genes involved in fermentation, drug transport, pathogenesis and response to oxidative stress. Genes related to cytotoxicity were differentially regulated but the gliotoxin biosynthesis genes were down regulated over the time course, while *Aspf1* was up-regulated at 9 h and 12 h. There was an up-regulation of genes in the subtelomeric regions of the genome as the interaction progressed. The genes up-regulated by iDC in the presence of *A. fumigatus* indicated that they were producing a pro-inflammatory response which was consistent with previous transcriptome studies of iDC interacting with *A. fumigatus* germ tubes. This study shows that *A. fumigatus* adapts to phagocytosis by iDCs by utilising genes that allow it to survive the interaction rather than just up-regulation of specific virulence genes.

## Introduction

The ascomycete fungus *Aspergillus fumigatus* (teleomorph *Neosartorya fumigata* [1]) is a saprophyte with a global distribution and is generally found in soil or decaying vegetation [2]. It produces vast numbers of asexual spores (conidia) which readily become airborne to aid dispersal. It is estimated that 200–300 airborne conidia are inhaled daily; their small size, *ca* 2.5–3.5 µm in diameter, allows them to enter the alveoli [3]. According to the immune status of the host *Aspergillus* spp are responsible for a spectrum of diseases in humans that range from the consequences of allergic host responses to often fatal invasive infection in the profoundly immunocompromised. For example, in patients with haematological malignancies who undergo allogeneic stem cell transplantation invasive aspergillosis (IA) is the leading infective cause of death [4].

Inhaled resting conidia that reach the alveoli of an immunocompetent host are inert to the immune system [5]. Upon germination they encounter a number of pulmonary defences including respiratory mucus, antimicrobial molecules, such as defensins, and pulmonary macrophages [6]. Macrophages recognise *A. fumigatus* through pathogen recognition receptors (PRR), the most important being Toll-like receptors (TLR 2 and TLR4 [7], [8]) and dectin-1 [9], [10]. Recognition of *A. fumigatus* by the innate immune system leads to the release of pro-inflammatory cytokines, such as TNF-α and IL8 [11], [12], [13], which is important for host defence.

The importance of a pro-inflammatory response in defence against *A. fumigatus* suggests a direct role for dendritic cells (DC), which act as a bridge between the innate and adaptive immune system [14]. DC exposed to germ tubes of *A. fumigatus in vitro* produce a pro-inflammatory response mediated by Dectin-1 [13]. A network of DCs is present in the lungs and at mucosal surfaces of most tissues where they sample their immediate microenvironment to detect pathogenic microbes. Pulmonary DCs phagocytose microbes and through cytokine signalling mature during migration to the lymph nodes and present microbial antigens to activate T-cells populations [15].

All soil fungi that cause disease in animals show a great degree of physiological adaptability, which is essential for their survival [16]. The ability to adapt to various nutrient sources and environmental conditions is a key factor in the ability of *A. fumigatus* to cause infection in humans [17]. There are increasing data indicating that basic metabolic processes in this fungus are more important for pathogenicity than *bona fide* virulence factors [16], [17], [18]. Although it produces toxins, their exact role in virulence remains unclear [19], whereas the ability to adapt to pH [20], iron limitation [21], and hypoxia [22] have been shown to be essential for virulence. There are limited reports on the transcriptional response of *Aspergillus* spp to the human immune system. Transcriptomic data have been obtained for *A. fumigatus* interacting with human neutrophils *in vitro* [23] and in a murine model of IA [14] showing that the fungus adapts to the nutritional challenges presented by the host. In this study we have investigated the response of *A. fumigatus* to monocyte-derived immature DC (iDC) over a 12 h time course to determine the dynamics of the transcriptional response of the fungus to a key component of the human innate immune system.

## Results

### Observations of the in vitro interaction between *A. fumigatus* and iDC

**Figure 1:**
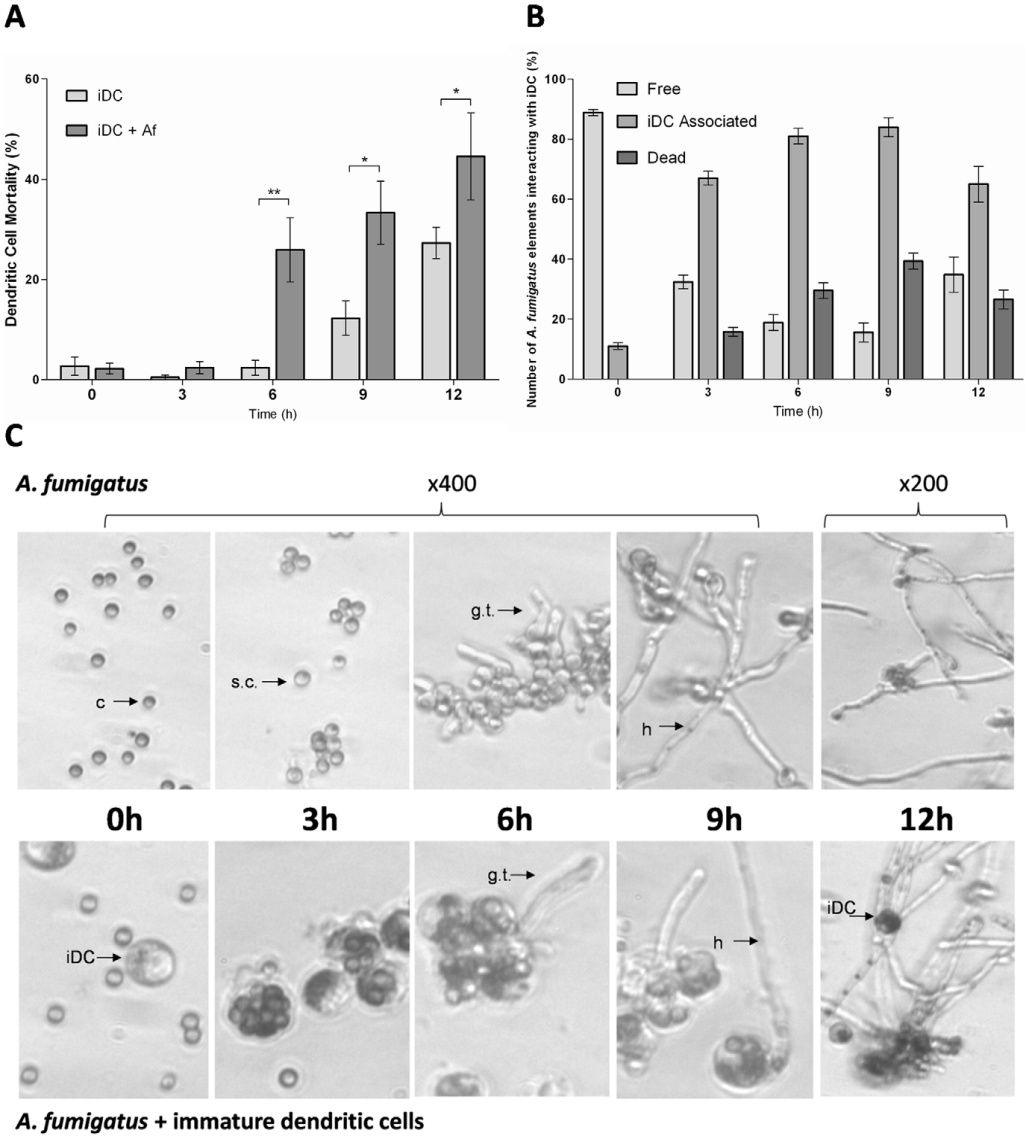
Interaction of iDC and *A. fumigatus in vitro*. Cell death of (a) immature Dendritic cells (iDC) asterisks indicate where there was a significant difference between treatments at each time point (t-test, p<0.05), and (b) *A. fumigatus* during their interaction over 12 h in RPMI medium incubated at 37°C and 5% CO2. iDC associated *A. fumigatus* included conidia that were ingested and those in direct physical contact with iDC. Both iDC and *A. fumigatus* were stained with neutral red for monitoring of viability by light microscopy. The viability of each was determined from the total number of cells / fungal elements observed at that time point. There were three replicate experiments and 300 cells over 10 fields of view were counted per time point, charts show the mean and standard errors. (c) Light micrographs of *A. fumigatus* developing from resting conidia to hyphae over 12 h in the presence or absence of iDCs; c – conidia, s.c. – swollen conidia, g.t. – germ tubes, h – hyphae, iDC – immature dendritic cells.

The increase in iDC cell death when grown in the presence or absence of *A. fumigatus* conidia is shown in Figure 1a. The significant increase in cell death at 6 h compared to 3h (ANOVA, Dunnett's post test p<0.01) in iDC infected with *A. fumigatus* may be attributable to the survival and development of phagocytosed conidia (Table 1). There was significantly more cell death in infected iDCs at 9 h and 12 h compared to uninfected iDCs (t-test, p<0.05); increased cell death was observed over the time course of the experiment in both treatments (ANOVA, Dunnett's post test with values compared to 0h, p<0.05). In uninfected iDC this was probably due to nutrient limitation or unfavourable culture conditions and was a contributing factor to the death of infected iDC at 9 h and 12 h (Fig. 1a). The majority of conidia were associated (in contact or phagocytosed) with iDC at 3 h and 6 h showing interaction between the cells. The growth medium and culture conditions had no effect on *A. fumigatus* viability but the interaction with iDCs did have a significant effect; up to 30% of the visible conidia were homogenously stained with neutral red by 6 h (Fig. 1b) indicating their non-viability. At 3 h and 6 h dead conidia were only observed in association with iDC, as the number of free conidia rose due to iDC lysis there were more dead conidia observed that were not attached to iDC at 9 h and 12 h (Fig. 1b). The sharp rise in the number of conidia associated with iDC at 3 h in this experiment (Fig. 1b and 1c) was consistent with the high capacity of iDCs to sample their surrounding microenvironment for potential pathogens through macropinocytosis, receptor mediated endocytosis and phagocytosis [24]. iDC constitutively express C-type lectin receptors, including Dectin-1 and DC-SIGN [25], that are involved in fungal recognition and phagocytosis [15]. 

**Table 1:** Development of *A. fumigatus* morphotypes interacting with iDCs in co-cultures over 12 h in RPMI medium (supplemented with 10% FCS).

*A. fumigatus* morphotypes (%)	3h	6h	9h	12h
Conidia	77±1.7	63.3±2	57.7±2.7	48.8±3.8
Swollen conidia	23±2	31.5±1.8	29.8±3.1	26.4±2.6
Germ Tubes	0	5.2±1.3	11.5±2.1	8.2±1.8
Hyphae	0	0	1±0.6	16.6±6

The counts derived from light microscopic observation of at least ten fields of view containing 200 cells at each time point; the total cell (fungal element) numbers included phagocytosed conidia. The numbers are mean ± standard error from three replicate experiments. Swollen conidia were distinguished by being large and appearing to having a thinner cell wall due to loss of the rodlet layer. Hyphae were distinguished from germ tubes by being longer in length >50 µm.

The surviving conidia interacting with iDCs developed at a rate consistent with those in medium alone (Fig. 1c), which was important for comparing gene expression in the presence/absence of iDCs. The number of free fungal cells increased with time due to the death of iDC and outgrowth of hyphae (Fig. 1b).

### FACS analysis of iDC maturation markers

**Figure 2:**
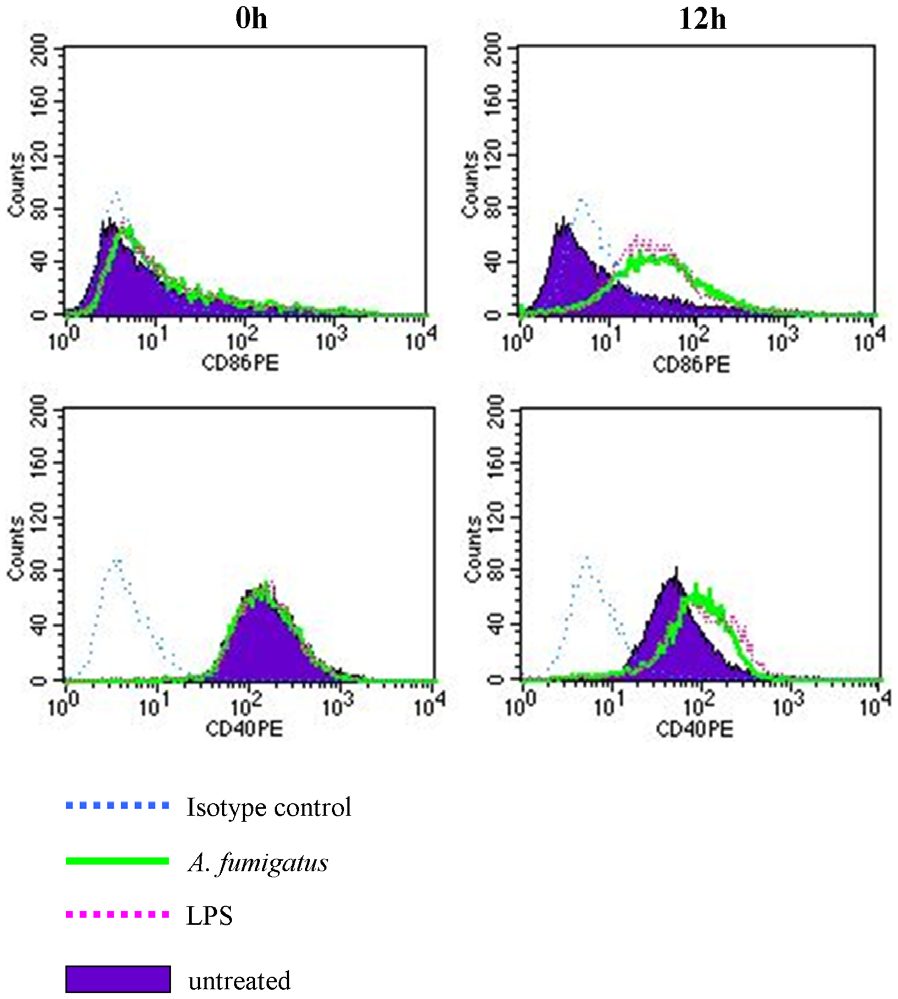
Expression of iDC maturation markers measured by FACS analysis at T = 0 h and 12 h. CD86 and CD40 show a shift from the control populations after T = 12 h similar to the effect induced by LPS.

The expression of iDC maturation markers confirmed the proper functioning of the cells. The flow cytometry data indicated the presentation of maturation markers on the surface of iDCs interacting with *A. fumigatus* (Fig. 2). CD86, essential for priming of T-cells against antigens, was observed at 12 h; CD40, an important protein for the activation of antigen-presenting cells was also observed. In a similar experiment by Gafa, *et al.*, both CD86 and CD83 were detected by FACS analysis 30 h after exposure to *A. fumigatus* [12].

### Gene expression in *A. fumigatus* interacting with iDC

**Figure 3:**
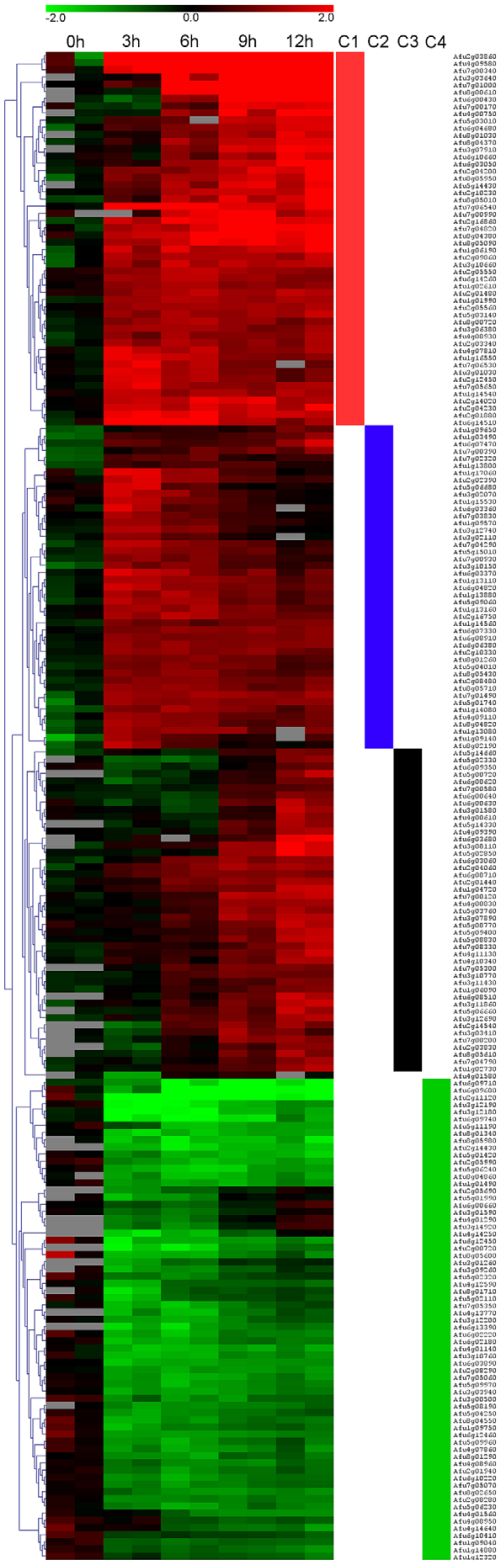
Genes differentially expressed by *A. fumigatus* interacting with iDC over 12 h *in vitro*. Gene expression heat map generated through comparison of *A. fumigatus* incubated with iDC to *A. fumigatus* incubated without iDC at each time point from 0 h to 12 h. Red indicates up-regulated, green indicates down-regulated. The red side bar indicates class I genes, blue for class II, black class III and green for class IV. Figure generated by hierarchical clustering (using the Euclidean distance and average linkage method) of genes identified as differentially regulated over time by SAM multi class analysis in TM4. The color of each spot (red, upregulated; green, downregulated) represents the log_2_-fold change in gene expression of *A. fumigatus* plus iDCs over *A. fumigatus* minus iDCs for the same time point.

Transcriptional profiles of *A. fumigatus* interacting with iDC over time were obtained by comparing the relative expression of *A. fumigatus* grown with or without iDC under the same growth conditions (37°C, 5% CO_2_ in RPMI medium for up to 12 h). The time points were selected based upon the time needed for *A. fumigatus* to develop distinct morphotypes. Significance analysis of microarrays (SAM multi-class analysis) using the TM4 microarray data analysis suite revealed 210 genes that were differentially regulated over the course of the experiment (Table S1). Hierarchical clustering (Euclidean distance and average linkage clustering, see Materials and Methods) applied to this dataset in TM4 revealed a temporal pattern of gene expression showing four distinct classes of differentially expressed genes (Fig. 3); class 1, genes up-regulated at all time points; class 2 genes up-regulated at 3 h and 6 h; class 3, genes up-regulated at 9 h and 12 h; class 4, genes down-regulated at all time points. The chromosomal locations of all the differentially expressed genes over the time course of the experiments are shown in Figure S1. Analysis of all the up-regulated genes by FunCat analysis [26] (Table 2) and a parallel Gene Ontology analysis [27] (Table S2) showed a similar emphasis on the following biological processes; transport, pathogenesis, and RNA processing. Ribosome biogenesis was also common to both but at a lower significance level in FunCat than GO. GO analysis indicated the importance of folic acid biosynthesis and para-aminobenzoic acid metabolism both of which have been described as essential for virulence [28]. Another difference between the analyses was the identification of the oxidation of fatty acids by FunCat. An advantage of FunCat was the greater number of genes assigned to functional categories than there were with GO analysis.

**Table 2:** Funcat Analysis (second level) of genes* up-regulated in *A. fumigatus* by exposure to iDC over the 12 h course of the experiment.

Transported Compounds - substrates (p:0.0016)		Cell Cycle (p: 0.0305)	
Gene ID	Gene Annotation	Gene ID	Gene Annotation
AFUA_2G03860,	plasma membrane low affinity zinc ion transporter	AFUA_2G05560	exonuclease
AFUA_3G03640	MFS siderochrome iron transporter MirB	AFUA_3G11860	microtubule associated protein EB1
AFUA_7G01000	aldehyde dehydrogenase	**Nucleotide/nucleoside/nucleobase binding (p: 0.042)**	
AFUA_2G16860	MFS multidrug transporter,	AFUA_4G08930	nucleolar GTPase
AFUA_1G06190	histone H4 arginine methyltransferase RmtA	AFUA_2G14020	ABC transporter
AFUA_2G09860	purine-cytosine permease	AFUA_6G08710	alkaline phosphatase
AFUA_8G00720	amino acid transporter	**Amino acid metab olism (p: 0.049)**	
AFUA_7G00390	MFS multidrug transporter	AFUA_7G01000	aldehyde dehydrogenase
AFUA_1G13800	MFS multidrug transporter	AFUA_2G04200	4-hydroxyphenylpyruvate dioxygenase
AFUA_3G12740	copper resistance-associated P-type ATPase	AFUA_1G06190	histone H4 arginine methyltransferase RmtA
AFUA_7G04290	amino acid permease (Gap1)	AFUA_5G03140	GTP cyclohydrolase
AFUA_5G15010	arsenite efflux transporter (ArsB)	AFUA_4G07810	L-serine dehydratase
AFUA_2G16750	nonsense-mediated mRNA decay protein 3	AFUA_2G12450	hydroxymethylglutaryl-CoA lyase
AFUA_8G05710	MFS sugar transporter Stl1	AFUA_2G04230	fumarylacetoacetate hydrolase FahA
AFUA_7G01490	MFS peptide transporter Ptr2	AFUA_5G06680	4-aminobutyrate transaminase GatA
AFUA_4G09110	cytochrome c peroxidase Ccp1	AFUA_1G13110	4-coumarate-CoA ligase
AFUA_5G14660	GABA permease	AFUA_5G01740	deoxyhypusine synthase
AFUA_6G00630	MFS transporter	AFUA_3G01580	GMC oxidoreductase
AFUA_3G01580	GMC oxidoreductase	AFUA_4G00610	aryl-alcohol dehydrogenase
AFUA_4G00610	aryl-alcohol dehydrogenase	AFUA_3G11430	Arginase
AFUA_5G14330	12-oxophytodienoate reductase	**Oxidation of fatty acids (p: 0.054)**	
AFUA_6G03060	MFS monosaccharide transporter	AFUA_1G14540	oxidoreductase, short-chain dehydrogenase/reductase family
AFUA_2G04060	NADH∶flavin oxidoreductase	AFUA_5G09400	carbonyl reductase
AFUA_2G01440	mitochondrial carrier protein	AFUA_3G03410	enoyl-CoA hydratase/isomerase family protein
AFUA_1G04720	C-8 sterol isomerase (Erg-1)	**Disease, virulence and defense (p: 0.072)**	
AFUA_4G00830	MFS peptide transporter	AFUA_7G01000	aldehyde dehydrogenase
AFUA_3G10770	RTA1 domain protein	AFUA_2G16860	MFS multidrug transporter
AFUA_1G02730	mitochondrial phosphate carrier protein (Ptp)	AFUA_1G06190	histone H4 arginine methyltransferase RmtA
**Protein Binding (p: 0.0158)**		AFUA_1G13800	MFS multidrug transporter
AFUA_1G06190,	histone H4 arginine methyltransferase RmtA	AFUA_5G15010	arsenite efflux transporter (ArsB)
AFUA_2G05550	conserved hypothetical protein	AFUA_7G00930	integral membrane protein
AFUA_1G01990,	ribosome biogenesis protein Rsa4	AFUA_6G04820	integral membrane protein , para-aminobenzoate synthase PabaA
AFUA_2G16750	nonsense-mediated mRNA decay protein 3	AFUA_6G00640	integral membrane protein
AFUA_8G04820,	ribonuclease P complex subunit Pop2	**Stress Response (p: 0.082)**	
AFUA_3G11860	microtubule associated protein EB1	AFUA_7G01000	aldehyde dehydrogenase
AFUA_1G06190,	histone H4 arginine methyltransferase RmtA	AFUA_4G09110	cytochrome c peroxidase Ccp1
**Phosphate metabolism (p: 0.0193)**		AFUA_6G08710	alkaline phosphatase
AFUA_1G02730	mitochondrial phosphate carrier protein (Ptp)	**Cellular signalling (p:0.088)**	
**Complex cofactor/cosubstrate/vitami n binding (p: 0.026)**		AFUA_2G10230	inositol oxygenase
AFUA_7G01000	aldehyde dehydrogenase	**DNA processing (p: 0.088)**	
AFUA_3G07910	UDP-glucose 4-epimerase	AFUA_7G03830	DNA repair protein Rad7
AFUA_2G10230	nositol oxygenase	AFUA_2G08480	ATP-dependent RNA helicase Mrh4
AFUA_1G16550	dihydrouridine synthase family protein	**RNA processing (p: 0.095)**	
AFUA_5G06680	4-aminobutyrate transaminase GatA	AFUA_2G05550	conserved hypothetical protein
AFUA_6G03370	short-chain dehydrogenase/reductase	AFUA_6G14260	U3 small nucleolar ribonucleoprotein protein Lcp5
AFUA_2G16750	nonsense-mediated mRNA decay protein 3	AFUA_1G01990	ribosome biogenesis protein Rsa4
AFUA_5G14330	12-oxophytodienoate reductase	AFUA_2G05560	exonuclease
AFUA_2G04060	NADH∶flavin oxidoreductase	AFUA_1G16550	dihydrouridine synthase family protein
AFUA_3G01580	GMC oxidoreductase	AFUA_6G08910	tRNA methyltransferase
AFUA_4G00610	aryl-alcohol dehydrogenase	AFUA_5G04010	tRNA-splicing endonuclease subunit Sen2
**Protein modification (p: 0.298)**		AFUA_8G05430	ribosome biogenesis protein Noc4
AFUA_1G14560	mannosidase MsdS	AFUA_8G04820	ribonuclease P complex subunit Pop2
AFUA_1G06190	histone H4 arginine methyltransferase RmtA		
AFUA_6G07330	methionine aminopeptidase, type I		

*Number of entries with annotations: 76 of 146 (52.05%).

p-value generated by Fisher's exact test indicating the significance of the number of hits for each category in the dataset compared to the hits per category in the whole genome.

The down-regulated genes were similarly analysed by both FunCat and GO analyses (Table 3 and S3). The following biological processes were significantly down-regulated; fermentation, sulphur metabolism, response to oxidative stress, TCA cycle, amino acid metabolism and transport.

**Table 3:** Funcat Analysis (second level) of genes* down-regulated in *A. fumigatus* by exposure to iDC over the course of the experiment.

Transported compounds (substrates) (p:00038)		C-compound and carbohydrate metabolism (p: 0.0114)	
Gene ID	Gene Annotation	Gene ID	Gene Annotation
AFUA_6G09710	MFS gliotoxin efflux transporter GliA	AFUA_8G01340	MFS sugar transporter
AFUA_8G01340	MFS sugar transporter	AFUA_5G06240	alcohol dehydrogenase
AFUA_2G00720	aldehyde dehydrogenase	AFUA_4G01290	endo-chitosanase
AFUA_3G12200	small oligopeptide transporter, OPT family	AFUA_6G12450	chaperone/heat shock protein Hsp12
AFUA_6G02220	MFS toxin efflux pump	AFUA_2G00720	aldehyde dehydrogenase
AFUA_4G01140	MFS multidrug transporter	AFUA_3G10760	phosphoketolase
AFUA_6G10220	small oligopeptide transporter, OPT family	AFUA_1G09750	aldehyde reductase (AKR1)
AFUA_7G05070	FAD dependent oxidoreductase	AFUA_7G05070	FAD dependent oxidoreductase
AFUA_8G02650	ABC multidrug transporter	AFUA_8G02650	ABC multidrug transporter
AFUA_5G06230	GABA permease	AFUA_2G08280	NADP-dependent malic enzyme MaeA
AFUA_4G01560	MFS myo-inositol transporter	AFUA_4G01560	MFS myo-inositol transporter
AFUA_4G14640	low affinity iron transporter	**Anaplerotic reactions (p: 0.017)**	
AFUA_6G10410	vacuolar protein sorting protein (VPS11)	AFUA_2G08280	NADP-dependent malic enzyme MaeA
**Detoxification (p: 0.00059)**		**Stress response (p: 0.0287)**	
AFUA_6G09710	MFS gliotoxin efflux transporter GliA	AFUA_6G12450	chaperone/heat shock protein Hsp12
AFUA_5G06240	alcohol dehydrogenase	AFUA_2G00720	aldehyde dehydrogenase
AFUA_2G00720	aldehyde dehydrogenase	AFUA_6G03890	spore-specific catalase CatA
AFUA_6G02220	MFS toxin efflux pump	AFUA_1G09750	aldehyde reductase (AKR1)
AFUA_4G01140	MFS multidrug transporter	AFUA_7G05070	FAD dependent oxidoreductase
AFUA_6G03890	spore-specific catalase CatA	AFUA_8G02650	ABC multidrug transporter
AFUA_8G02650	ABC multidrug transporter	**Tricarboxylic-acid pathway (citrate cycle, Krebs cycle, TCA cycle) (p: 0.031)**	
**Nitrogen, sulfur and selenium metabolism (p: 0.00064)**		AFUA_7G05070	FAD dependent oxidoreductase
AFUA_2G00720	aldehyde dehydrogenase	AFUA_2G08280	NADP-dependent malic enzyme MaeA
AFUA_3G12200	small oligopeptide transporter, OPT family	**Electron transport and membrane-associated energy conservation (p: 0.0341)**	
AFUA_5G04250	homocysteine synthase CysD	AFUA_2G00720	aldehyde dehydrogenase
AFUA_6G10220	small oligopeptide transporter, OPT family	AFUA_7G05070	FAD dependent oxidoreductase
AFUA_5G06230	GABA permease	AFUA_2G08280	NADP-dependent malic enzyme MaeA
AFUA_1G14880	N-acylethanolamine amidohydrolase	**Disease, virulence and defense (p: 0.0422)**	
**Fermentation (p: 0.0026)**		AFUA_6G09710	MFS gliotoxin efflux transporter GliA
AFUA_5G06240	alcohol dehydrogenase	AFUA_2G00720	aldehyde dehydrogenase
AFUA_2G00720	aldehyde dehydrogenase	AFUA_6G02220	MFS toxin efflux pump
AFUA_3G10760	phosphoketolase	AFUA_8G02650	ABC multidrug transporter
AFUA_1G09750	aldehyde reductase (AKR1)	**Amino acid metabolism (p: 0.0824)**	
**Complex cofactor/cosubstrate/vitamine binding (p: 0.0068)**		AFUA_2G00720	aldehyde dehydrogenase
AFUA_2G00720	aldehyde dehydrogenase	AFUA_5G04250	homocysteine synthase CysD
AFUA_6G03890	spore-specific catalase CatA	AFUA_1G09750	aldehyde reductase (AKR1)
AFUA_5G04250	homocysteine synthase CysD	AFUA_7G05070	FAD dependent oxidoreductase
AFUA_1G09750	aldehyde reductase (AKR1)	AFUA_2G08280	NADP-dependent malic enzyme MaeA
AFUA_7G05070	FAD dependent oxidoreductase	**Cellular sensing and response to external stimulus (p: 0.096)**	
AFUA_2G08280	NADP-dependent malic enzyme MaeA	AFUA_6G12450	chaperone/heat shock protein Hsp12
	AFUA_3G12200	small oligopeptide transporter, OPT family	
		AFUA_1G09750	aldehyde reductase (AKR1)
		AFUA_6G10220	small oligopeptide transporter, OPT family

*Number of entries with annotations: 24 of 68 (35.29%).

p-value generated by Fisher's exact test indicating the significance of the number of hits for each category in the dataset compared to the hits per category in the whole genome.

FunCat analysis of the genes in class 2 (up-regulated at 3 h and 6 h) showed genes mainly involved in metabolism and transport (Table 4), whereas the genes of class 3 (up-regulated at 6 h and 9 h) displayed a greater diversity of functions and were involved in secondary metabolism, oxidation of fatty acids, transport and nutritional response (Table 5).

**Table 4:** Funcat Analysis (third level) of temporally regulated genes*, class 2, in *A. fumigatus* by exposure to iDC over the course of the experiment.

Membrane lipid metabolism (p: 0.018)		Detoxification by export (p: 0.10)	
Gene ID	Gene Annotation	Gene ID	Gene Annotation
AFUA_6G03370	short-chain dehydrogenase/reductase	AFUA_7G00390	MFS multidrug transporter
**Isoprenoid metabolism (p: 0.018)**		AFUA_1G13800	MFS multidrug transporter
AFUA_1G13160	geranylgeranyl diphosphate synthase	**RNA binding (p: 0.11)**	
**tRNA processing (p: 0.0185)**		AFUA_2G16750	nonsense-mediated mRNA decay protein 3
AFUA_5G04010	tRNA-splicing endonuclease subunit Sen2	AFUA_8G05430	ribosome biogenesis protein Noc4
AFUA_8G04820	ribonuclease P complex subunit Pop2	AFUA_8G04820	ribonuclease P complex subunit Pop2
**Resistance proteins (p: 0.041)**		**Ion Transport (p: 0.11)**	
AFUA_1G13800	MFS multidrug transporter	AFUA_3G12740	copper resistance-associated P-type ATPase
AFUA_5G15010	arsenite efflux transporter (ArsB)	AFUA_7G04290	amino acid permease (Gap1)
AFUA_6G04820	para-aminobenzoate synthase PabaA	AFUA_5G15010	arsenite efflux transporter (ArsB)
**Transport Routes (p: 0.046)**		**Peptide transport (p: 0.12)**	
AFUA_7G01490	MFS peptide transporter Ptr2	AFUA_7G01490	MFS peptide transporter Ptr2
**rRNA processing (p: 0.06)**			
AFUA_6G08910	tRNA methyltransferase		
AFUA_8G05430	ribosome biogenesis protein Noc4		
AFUA_8G04820	ribonuclease P complex subunit Pop2		

*Number of entries with annotations: 27 of 45 (60.00%).

p-value generated by Fisher's exact test indicating the significance of the number of hits for each category in the dataset compared to the hits per category in the whole genome.

**Table 5:** Funcat Analysis (third level) of temporally regulated genes*, class 3, in *A. fumigatus* by exposure to iDC over the course of the experiment.

FAD/FMN binding (p: 0.00032)		Cellular import (p: 0.072)	
Gene ID	Gene Annotation	Gene ID	Gene Annotation
AFUA_3G01580	GMC oxidoreductase	AFUA_5G14660	GABA permease
AFUA_4G00610	aryl-alcohol dehydrogenase	AFUA_6G00630	MFS transporter
AFUA_5G14330	12-oxophytodienoate reductase	AFUA_6G03060	MFS monosaccharide transporter
AFUA_2G04060	NADH∶flavin oxidoreductase	AFUA_4G00830	MFS peptide transporter
**Lipid/fatty acid transport (p: 0.089)**		**Transport facilities (p: 0.078)**	
AFUA_2G01440	mitochondrial carrier protein	AFUA_6G00630	MFS transporter
AFUA_1G04720	C-8 sterol isomerase (Erg-1)	AFUA_6G03060	MFS monosaccharide transporter
AFUA_3G10770	RTA1 domain protein	AFUA_2G01440	mitochondrial carrier protein
**Electron transport (p: 0.0205)**		AFUA_4G00830	MFS peptide transporter
AFUA_3G01580	GMC oxidoreductase	AFUA_1G02730	mitochondrial phosphate carrier protein (Ptp)
AFUA_4G00610	aryl-alcohol dehydrogenase	**Mitochondrial transport (p: 0.0906)**	
AFUA_5G14330	12-oxophytodienoate reductase	AFUA_2G01440	mitochondrial carrier protein
AFUA_2G04060	NADH∶flavin oxidoreductase	AFUA_1G02730	mitochondrial phosphate carrier protein (Ptp)
**Oxidation of fatty acids (p: 0.023)**		**Peptide transport (p: 0.0953)**	
AFUA_5G09400	carbonyl reductase	AFUA_4G00830	MFS peptide transporter
AFUA_3G03410	enoyl-CoA hydratase	**Neurotransmitter transport (p: 0.099)**	
**Metabolism of secondary products derived from primary amino acids (p: 0.049)**		AFUA_5G14660	GABA permease
AFUA_3G01580	GMC oxidoreductase	**Carrier (electrochemical potential-driven transport) (p: 0.099)**	
AFUA_4G00610	aryl-alcohol dehydrogenase	AFUA_1G02730	mitochondrial phosphate carrier protein (Ptp)
**Energy conversion and regeneration (p: 0.054)**		**Homeostasis of anions (p: 0.099)**	
AFUA_5G14330	12-oxophytodienoate reductase	AFUA_1G02730	mitochondrial phosphate carrier protein (Ptp)
AFUA_2G04060	NADH∶flavin oxidoreductase	**Perception of nutrients and nutritional adaptation (p: 0.099)**	
**Isoprenoid metabolism (p:0.063)**		AFUA_6G03060	MFS monosaccharide transporter
AFUA_1G04720	C-8 sterol isomerase (Erg-1)	**Nutrient starvation response (p: 0.11)**	
		AFUA_6G08710	alkaline phosphatase

*Number of genes with annotations: 21 of 45 (46.67%).

p-value generated by Fisher's exact test indicating the significance of the number of hits for each category in the dataset compared to the hits per category in the whole genome.

A closer examination of pathogenicity related genes (Table 6) indicated that the gliotoxin gene cluster was not important for the interaction with iDC. The response to oxidative stress and oxygen radical detoxification was important including up-regulation of the MnSOD, *AfSOD3* (Afu1g14550) and genes involved in pyomelanin biosynthesis (Afu2g04230 and Afu2g04200). The only toxin that was differentially regulated was *Aspf1* (Afu5g02330), at 9 h and 12 h. The pathogenicity related genes expressed suggest a defensive response from the fungus aimed at surviving the interaction with iDC.

**Table 6:** Relative expression of pathogenicity related genes by *A. fumigatus* in co-incubation with iDC.

ORF	Gene Name	Expression Data (Log_2_)			
		3h	6h	9h	12 h
***Allergens***					
Afu5g02330	major allergen and cytotoxin *Aspf1*	−0.72	−0.37	0.12	1.09
Afu4g09580	major allergen *Aspf2*	3.62	3.26	3.24	3.6
Afu2g03830	allergen *Aspf4*	−0.67	0.115	0.59	1.1
***Gliotoxin Cluster***					
Afu6g09680	O-methyltransferase *GliM*	−1.10	−2.3	−2.39	−2.26
Afu6g09740	thioredoxin reductase *GliT*	−1.89	−2.1	−1.78	−1.15
Afu6g09710	MFS gliotoxin efflux transporter *GliA*	−1.12	−1.9	−2.1	−1.98
Afu6g09730	cytochrome P450 oxidoreductase *GliF*	−1.86	−1.76	−1.46	−1.22
***Tyrosine catabolism/Pyomelanin Synthesis***					
Afu2g04230	fumarylacetoacetate hydrolase *FahA*	1.68	1.6	1.6	1.74
Afu2g04220	homogentisate 1,2-dioxygenase (*HmgA*), putative	1.23	1.03	1.23	1.4
Afu2g04210	Conserved protein	0.8	0.72	1.2	1.32
Afu2g04200	4-hydroxyphenylpyruvate dioxygenase, putative	1.02	1.1	1.66	2.2
***Response to Oxidative Stress***					
Afu6g12450	chaperone/heat shock protein Awh11	−1.62	−1.35	−1.2	−1.07
Afu6g03890	spore-specific catalase *CatA*	−1.5	−1.31	−1.18	−1.12
Afu4g09110	cytochrome c peroxidase *Ccp1*, putative	1.22	1.06	0.77	0.81
Afu1g14550	Mn superoxide dismutase *MnSOD*	2.1	1.03	1.18	1.46
Afu6g04820	para-aminobenzoate synthase *PabaA*	1.57	1.23	0.98	0.81
Afu7g00170	dimethylallyl tryptophan synthase	−0.46	0.62	1.86	1.77
Afu7g05060	MgtC/SapB family membrane protein	−1.18	−1.42	−1.18	−1.03

### Gene expression of iDC interacting with *A. fumigatus*

Analysis of gene expression in iDCs interacting with *A. fumigatus* showed differential expression of 16 genes, 14 up-regulated and 2 down-regulated, from 117 genes on the immune arrays (Table 7). Six of the genes were continuously up-regulated throughout the experiment with 8 showing up-regulation over its time course. The down-regulated genes, *DC-SIGN* and *TNFRSF1*, showed reduced expression at 12 h.

**Table 7:** Relative expression of immune-related genes by iDCs in co-culture with *A. fumigatus* over 12 h in RPMI medium (supplemented with 10% FCS).

Gene	Gene Function	Log_2_ ratio of differential regulation			
		3 h	6h	9h	12h
**CCL4**	Chemokine activity	3.45	3.46	3.52	3.51
**CCL5**	Chemokine activity	-	1.68	2.05	2.16
**CXCL1**	Chemokine activity	-	-	-	1.02
**CXCL2**	Chemokine activity	3.49	4.10	4.15	4.25
**CXCL3**	Chemokine activity	-	-	1.11	1.29
**CCL20**	Chemokine activity	-	1.79	2.51	2.84
**CSF2**	Granulocyte macrophage colony-stimulating factor receptor binding	-	-	1.67	2.05
**IL1A**	Cytokine activity	-	1.24	1.53	1.76
**IL1B**	Interleukin-1 receptor binding	3.09	3.35	3.32	3.17
**IL1R2**	Immune Response	1.55	-	-	-
**IL8**	Chemokine activity	1.23	1.78	2.20	2.30
**NFKBIA**	Ubiquitin protein ligase binding	2.08	2.49	2.42	2.46
**TXN**	Protein binding	-	-	1.32	1.23
**TNF**	Inflammatory response	1.40	1.99	2.30	2.28
**DCSIGN**	Immune Response	-	-	−1.03	−1.19
**TNFRSF1**	Inflammatory Response	-	-	−1.00	−1.15

Spaces indicate that there was no significant differential expression at that time point.

The increased expression of Th1 cytokines, *IL1α*, *IL1β* and *TNF* indicated a pro-inflammatory response which was consistent with previous results for *A. fumigatus* interacting with iDCs [13] and monocytes [11], [29]. Increased expression of *IL1α*, *IL1β*, *TNF*, *IL8* and *CCL20* was common to each interaction study. The up-regulation of *TXN* has also been associated with a Th1 response [30]. There was increased expression of genes, *CXCL1*, *CXCL3*, *CSF2*, that encode proteins chemoattractant for neutrophils and macrophages at later time points, which might be related to iDC maturation or emergence of germ tubes.

**Figure 4:**
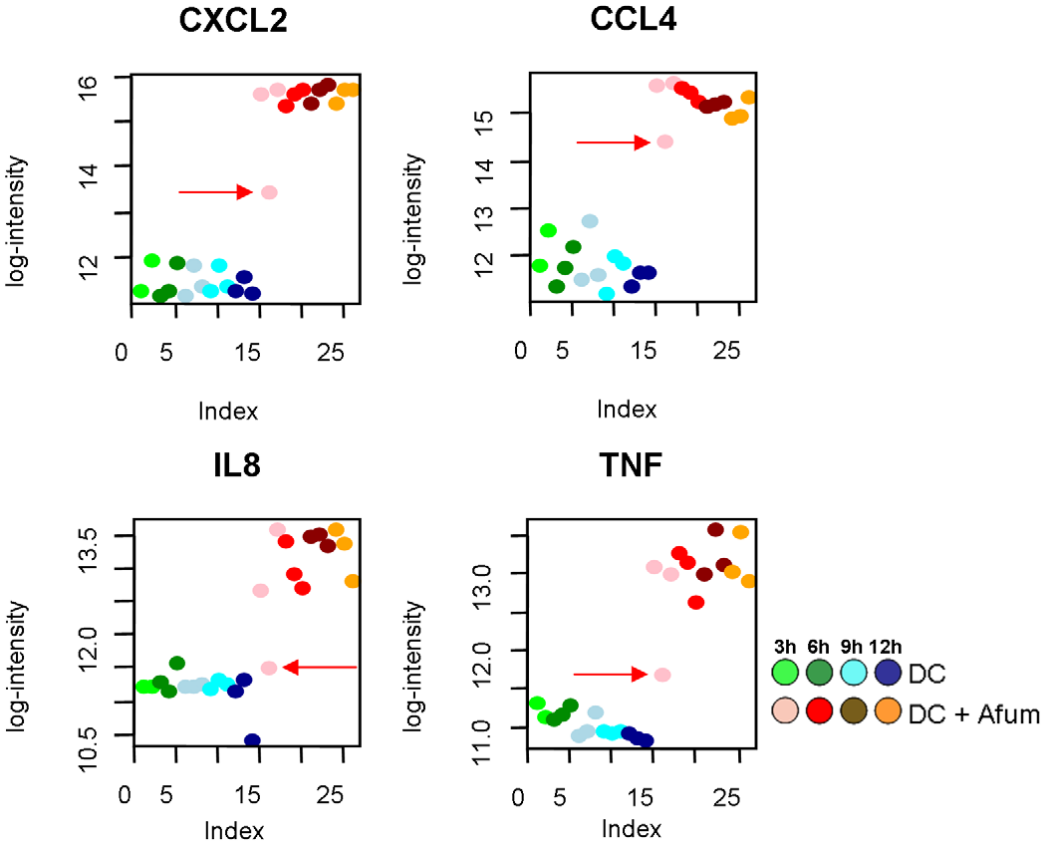
Scatter plots of genes differentially expressed by iDCs in the presences or absence of *A. fumigatus* over 12 h. The plots show differences between donors with regard to the induction of gene expression in genes important for immunity to fungi. The warm colours represent the response of iDC with *A. fumigatus*, the cold colours show gene expression in the absence of *A. fumigatus*. Red arrows indicate the donor outlier in the datasets.

Scatter plots of gene expression showing each donor and each time point indicated variability in the response of one of the donors (Fig. 4). The greatest variability in donor response was observed in the 3 h expression data for *TNF*, *CCL4*, *CXCL2*, *IL8* and *IL1β*; this time delay could have an effect on the host's ability to mount an effective response.

### RT-qPCR validation of array data

**Figure 5:**
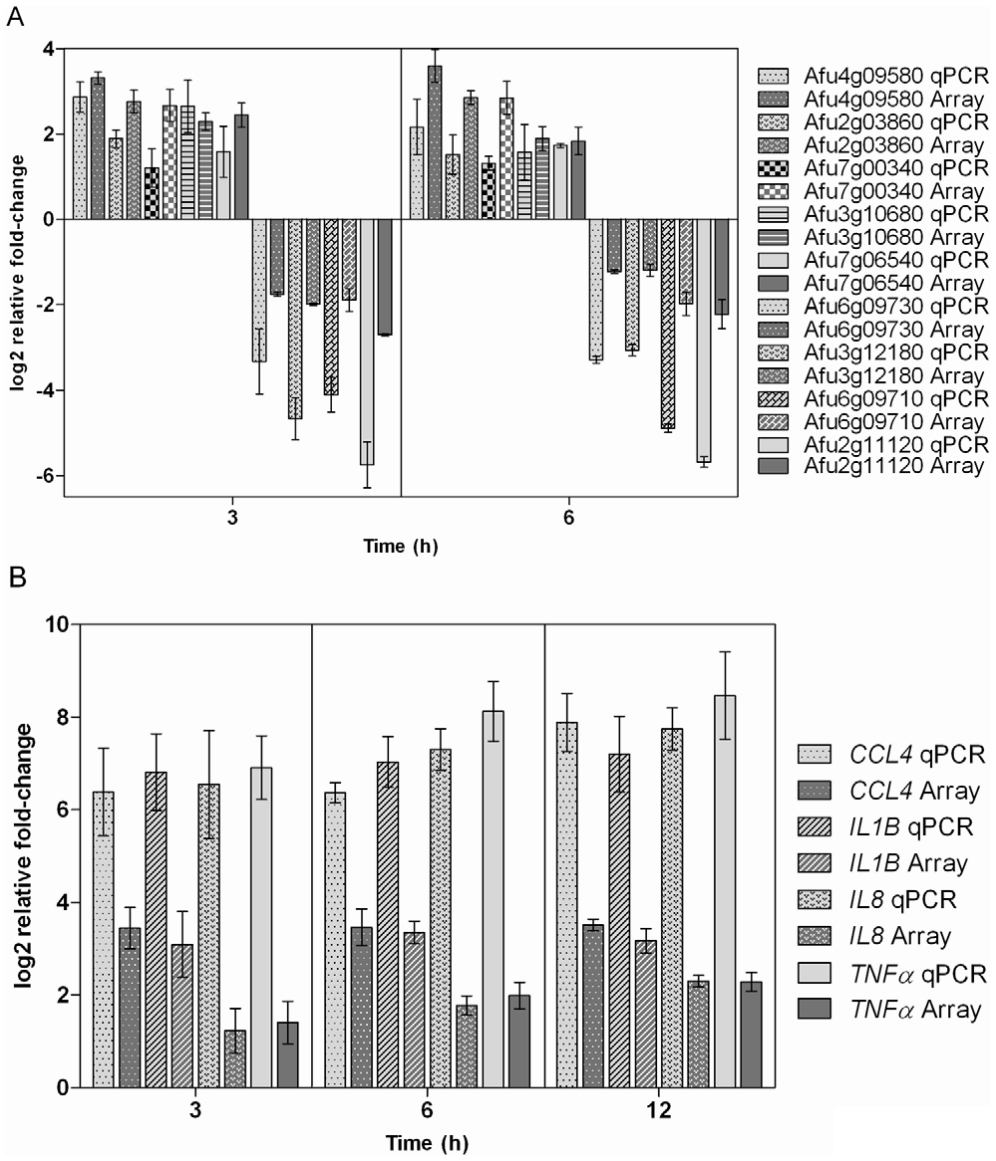
RT-qPCR validation of differential gene expression observed in microarray experiments. (a) Gene expression data from the *A. fumigatus* microarray shown as mean log_2_ values of ratios of relative expression determined by RT-qPCR compared to array data. (b) Gene expression data from the immune gene microarray shown as mean log_2_ values of ratios of relative expression determined by RT-qPCR compared to array data. Error bars in both charts indicate the standard error.

PCR results confirmed the differential expression of selected genes from the micro-array analyses (Fig. 5a and 5b); genes tested from both arrays show the same expression patterns for both qRT-PCR and microarray. These genes were selected from a list of genes that showed the greatest differential expression on the array.

## Discussion

### *Aspergillus* infection of iDC

During the interaction with *A. fumigatus* there was increase in iDC cell death at 6h and 9h (Fig. 1a). Phagocytic cells interacting with other pathogens are known to undergo programmed cell death (PCD) to control the spread of the infectious agent [31]. An immune evasion strategy for some microbes is the induction of PCD in iDCs, *Salmonella* and *Shigella* have been shown to activate caspases in iDCs leading to PCD [32]. In this study *A. fumigatus* may have actively contributed to iDC cell death through secreted molecules. The toxin Aspf1 can induce PCD in iDCs *in vitro* [33] and it has been reported that gliotoxin can induce PCD in monocytes and iDCs through the proapoptotic mitochondrial protein Bak leading to caspase-3 activation [34], [35]. Gliotoxin is the most abundant toxin produced by *A. fumigatus* and is released by hyphae [34], this could explain the increase in iDC cell death after 6 h in this study (Fig. 1a) since hyphae were formed after 6 h. However, the genes necessary for gliotoxin biosynthesis were down regulated (Table 6); suggesting that for gliotoxin to be the primary means of fungus-induced cell death in this interaction would require that basal transcription levels be sufficient to induce cytotoxic effects in APCs. Interestingly, the amount of gliotoxin found to be inhibitory to APCs (35 ng/ml) is lower than the amounts found in the serum of patients with IA suggesting that it is active at low concentrations [34]. The transcriptional response of the fungus (Table 6) did not suggest that it was producing a battery of toxins to cause iDC cell death but transcription may not tell the whole story. Conidia contain stocks of preformed proteins that can enhance competitiveness in new micro-environments. A study of the proteome of conidia revealed that they contain relatively high amounts of proteins for oxidative stress response, allergens and the toxin Asp-haemolysin [36] which may be important for interaction with human host cells. Asp-haemolysin is a member of the aegerolysin protein family which has cause cytoxicity by interacting with cell membranes [37]. Asp- haemolysin has cytolytic activity against neutrophils and macrophages [38] indicating that it may have a role during interactions with host cells.

The amount of fungal killing by iDCs was less than that observed with neutrophils at a lower MOI [23]; neutrophils at an MOI of 1 caused *ca* 50% decrease in viable conidia after 2 h exposure. All the dead conidia were associated (phagocytosed or in physical contact) with iDC; by 3 h 68% of conidia were associated with iDCs rising to 85% at 9 h (Fig. 1b). Similar levels of phagocytosis were reported by Gafa, *et al.*, where 30% of conidia were phagocytosed after 30 min and 48% after 2 h at an MOI of 1 [12]. Delayed killing of microbes has been observed in iDC [15] and this may be due to a need to preserve antigens for presentation. The phagosome of iDCs maintains a neutral to alkaline pH to regulate the activity of proteolytic enzymes which is in contrast to neutrophils and macrophages which acidify the phagosome to activate enzymes such as cathepsin [39]. NADPH oxidase plays a role in protease activation [39] and is expressed in iDC but at much lower levels than in neutrophils; its role in iDCs is to generate ROS to regulate the pH of the phagosome over several hours to prevent antigen degradation rather than generating an oxidative burst [40]. This reduced protease activity and low level ROS production may contribute to the survival of conidia in the iDC phagosome. Differences in the survival time of conidia compared to hyphae ingested by iDC have been reported [15], furthermore, it was found that conidia and germ tubes were ingested by different mechanisms of phagocytosis [15], which may be due to recognition of antigens on the hyphal surface that are obscured by the hydrophobin layer of conidia.

Ingestion and processing of conidia by iDC involves the expression of dendritic cell-specific intercellular adhesion molecule-3-grabbing nonintegrin (DC-SIGN) this protein is a type II C-type lectin that functions as an adhesion receptor [41]. In this study its expression by iDC decreased at 9 h and 12 h (Table 7), at 9 h there was no great increase in the number of iDC associated conidia and at 12 h there was a decrease in the number of iDC associated conidia (Fig. 1b). As iDC mature there is a decrease in their uptake of pathogens and decreased expression of pathogen recognition genes [42], [43] these would explain the reduced DC-SIGN expression and is supported by the presentation of maturation markers at 12 h (Fig. 2).

During the generation of monocyte derived iDC the monocytes were exposed to IL4, which is necessary for DC-SIGN expression [44]. It has been suggested that IL4 is an important factor in the susceptibility to *A. fumigatus* by encouraging a Th2 response [44]. This initial priming with IL4 may explain the relative persistence of *A. fumigatus* conidia in this study since the initial uptake of conidia would be governed by the Th2 environment leading to reduced microbicidal activity [44]. As the time course progressed the iDC were exposed to cell wall elements from developing fungi (swollen conidia and germ tubes) that could be recognised by other cell surface receptors such as TLR2 and Dectin-1 leading to a proinflammatory response [45] indicated by the gene expression data (Table 7).

### Temporal Gene Expression in *A. fumigatus* interacting with iDC

There was a clear indication that *A. fumigatus* alters its pattern of gene expression over time when interacting with iDC (Fig. 3). A bias towards subtelomeric gene expression has been reported during infection in the murine lung and exposure to neutrophils *in vitro* [14]. In this study differential expression of genes in subtelomeric regions was observed, particularly the subtelomeric region of chromosome 7 (Fig. S1). This may be related to the ability of *A. fumigatus* to survive interactions with immune cells as indicated by the expression of efflux pumps and the allergen *Aspf2* (Table S4).

Interaction with iDCs induces a number of transcriptional responses in *A. fumigatus*. Analysis of the genes up-regulated over the course of the experiment (Tables 3 and S2) indicated increased transport of amino acids, purines, pyrimidines, amino acid and vitamin biosynthesis, which suggested the involvement of the cross-pathway control (CPC) regulatory system. In fungi, this type of eIF2α kinase signalling cascade creates a transcriptional response upon conditions of nutritional stress, starvation for amino acids being the most prominent one, to result in increased expression of numerous biosynthetic genes. Deletion of the gene encoding the CPC transcriptional regulator CpcA in *A. fumigatus* results in attenuated virulence in a mouse model of IA [46]. However, additional data indicate that basal but not elevated expression of CpcA is required for full pathogenicity [47], [48]. In our data set, the *cpcA* transcript itself was not differentially regulated during the experimental time course, which indicated the absence of a genuine cross-pathway control response. Furthermore, comparison of the categorised transcripts with profiling data generated from *A. fumigatus* after histidine starvation, a validated trigger for the CPC, revealed only limited overlap with differentially regulated genes in this study (unpublished data). Accordingly, our profiles indicate pathway-specific responses rather than a comprehensive derepression of the cross-pathway control system.

The class 2 genes (Table 4), showed an increase in genes necessary for detoxification and resistance through efflux, which was also observed in the early transcriptional response of conidia emerging from dormancy [49]. The genes with later up-regulation, class 3 (Table 5), showed a change in emphasis to fatty acid transport and oxidation. This is consistent with the fungus utilising fatty acids as a major carbon source and has been observed in the interaction of *A. fumigatus* with human neutrophils [23] and *in vivo* in mouse lungs [14]. Germinating conidia interacting with immune cells showed decreased expression in genes for aerobic respiration (Table 3) which is in contrast to other work that has examined the transcriptome of conidia emerging from dormancy [49]. These point to metabolic adaptations in conidia during germination informed by their microenvironment. There was continued evidence of amino acid degradation with metabolism of derivatives of primary amino acids. This has been reported to be important for pathogenesis since deletion of methylcitrate synthase had a negative impact on pathogenicity [18], [50]. There was differential regulation of genes in the tyrosine degradation pathway; this pathway consists of a cluster of six genes, four of which (AFUA2g04200 (*hppD*), AFUA_2g4210, AFUA_2g04220 (*hmgA*), AFUA_2g04230 (*fahA*)) were up-regulated during the interaction between *A. fumigatus* and iDC. The up-regulation of *fahA* is an indicator that it was acting on 4-fumaryl-acetoacetate to produce fumarate (for TCA cycle) and acetoacetate (acetyl CoA formation) for primary metabolic processes due to the prevailing nutrient availability.

Up-regulation of genes involved in nutrient perception and nutritional adaptation (AFUA_8g00720, AFUA-8g05710, AFUA_6g03060) and starvation response (AFUA_6g08710) was observed as the experiment progressed towards 12 h. AFUA_8g05710 is an orthologue of yeast STl1 which is involved in the reposnse to osmotic stress and glycerol transport [51]. AFUA-8g05710 is an amino acid transporter that is putatively involved in the synthesis of an ETP-type (epipolythiodioxopiperazine) molecule [52] and AFUA_6g03060 is an MFS monosaccharide transporter. This could be a response to the hyphae emerging from iDC and adjusting to the need to obtain nutrients from the growth medium. AFUA-6g08710 is an alkaline phosphatase that is regulated by the PHO80 homologue PhoB [53] as part of the phosphate acquisition pathway. Its regulation in this experimental system may represent part of the nutritional adaptation necessary to acquire inorganic phosphorous from the growth medium after emergence from iDC rather than indicating a strict starvation response since the growth medium was nutrient replete.

### Comparison with other Array Analyses

The temporally regulated genes from this study were compared to gene expression datasets created by McDonagh, *et al.* [14] of *A. fumigatus* transcriptomes during incubation in murine lungs and with neutrophils. This revealed some interesting similarities with each study showing regulation of *AfSOD3* (Afu1g14550), *hppD*, (Afu2g04200), *mirB* (Afu3g03640) and *ald4* (Afu7g01000). In each dataset there was a response to oxidative stress, and significant differential expression of genes involved in transport, fatty acid metabolism and aerobic respiration. There was an increase in genes involved in nutrient starvation response (FunCat p-value 0.11) and perception of nutrients (p-value 0.09), which was an important component of the response to the *in vivo* environment [14]. Differences between expression in co-culture with iDC and *in vivo* in murine lungs were up-regulation of ribosome biogenesis, RNA processing, down-regulation of amino acid metabolism, and no differential expression of siderophore-iron transport.

### Response to Oxidative Stress

The response to oxidative stress is important for pathogenic fungi interacting with the human immune system. Genes involved in the oxidative stress response are both up and down regulated in interaction with iDC. Cytochrome C peroxidase (AFUA_4g09110) was up-regulated during the first 6 h of the interaction along with *rad7*, (AFUA_7g03832) which is involved in repair of UV damage and may also be required for oxidative stress response. *AfSOD3* (Aspf6) was up-regulated at all time points. It has been shown that *A. fumigatus* SOD enzymes are internal, cytoplasmic or mitochondrial, and may play a role in detoxification of fungal derived oxidants rather than having a role in detoxifying host derived reactive oxygen species [54]. An interesting observation was that no catalases were up-regulated and *catA*, spore-specific catalase, (AFUA_6g03890), was down-regulated during the interaction with iDC which was to be expected after germination. The absence of differential catalase expression during interaction with iDCs contrasts with the up-regulation of *cat2* (AFUA_8g016760) during the interaction with neutrophils [14], [23] and in murine lungs [14]. This may be a characteristic of the response to the ROS burst produced by neutrophils, which is of reduced importance to the antimicrobial repertoire of dendritic cells. Previous studies have shown that deletion mutants for *catA* and *cat2* showed similar to wild type sensitivity to killing by neutrophils [55]; mycelial cat 1 and *cat2* bestow limited protection from host immune cells [56].

Another potential defence against ROS is melanin. The production of pyomelanin has been shown in *A. fumigatus* as a by-product of the tyrosine degradation pathway [57] which showed up-regulation during the *A. fumigatus* and iDC interaction. Homogentisate (HGA) synthesis is catalysed by *hppD*; if there is an imbalance between *hppD* and *hmgA* activity there can be an accumulation of HGA leading to pyomelanin formation [58]. The expression of *hppD* increased relative to *hmgA* during the course of the interaction with iDC (Table 6) and could have led to pyomelanin formation. Melanin formation in hyphae has been reported [59], [60] and up-regulation of *hppD* has been observed during interaction with iDC (Table 6), neutrophils [14] and in murine lung [14], which may suggest that pyomelanin formation is part of the *A. fumigatus* stress response. However, pyomelanin production appears to be associated with cell wall stress rather than oxidative stress [58]. Increasingly there is evidence that non-oxidative mechanisms are employed by immune cells interacting with fungi [54] and the fungal cell wall would be a primary target. Melanin has a role in the structure and stability of the conidial cell wall [61] and it is conceivable that hyphal melanin may be involved in maintaining cell wall stability in response to stress.

### Iron Acquisition

Another difference between the interactions of *A. fumigatus in vivo* and with iDCs is the regulation of genes involved in iron acquisition. Only *mirB* (AFUA_3g03640) was differentially regulated in response to iDCs whereas eleven iron acquisition genes were differentially regulated in murine lungs [14]. This may be explained by the artificial nature of cell interaction studies where the growth medium is iron replete unlike *in vivo* where iron is actively sequestered as a nutritional defence by the host.

### Toxins

Of 77 gene clusters identified during interaction in murine lung [14], 21 of these showed genes with differential regulation during interaction with iDC (Fig. S1); eight of these genes clusters were located in subtelomeric regions of the genome (Table S4). During the interaction of *A. fumigatus* with iDCs the gene cluster for biosynthesis of gliotoxin was down-regulated (Table 6). Gliotoxin has been reported to have a role in pathogenicity but its significance in pathogenicity is dependent on the type of host immunosuppression [19]. Both the gliotoxin and pseurotin gene clusters showed increased expression during interaction in the murine lung [14] but this was not observed in the interaction with iDCs. This further suggests that genes that are important for establishment of infection in the lung may not be required for interaction with immune cell subsets *in vitro*. The one toxin gene that was clearly up-regulated during the course of the experiment was *Aspf1* (Table 6), a ribotoxin that cleaves a phosphodiester bond in the 28S ribosomal RNA causing inhibition of protein synthesis and death by apoptosis [62], [63]. Its role in iDC cell death was unclear from this experiment but its increased expression at 12 h occurred after the largest increase in *A. fumigatus*-related iDC cell death (Fig. 1a). The gene AFUA_7g00170 encodes a dimethylallyl tryptophan synthase (DMAT), these enzymes have been implicated in ergot alkaloid, *e.g.* fumigaclavine C, synthesis [64]. Its regulation resembles that of *Aspf1* and may be another indicator that the fungus was shifting its metabolism to respond to confrontation by iDC.

### Interaction between iDC and *A. fumigatus*

iDCs play a crucial role in the defence against *A. fumigatus*. First, they mount an immediate immune response to fungi by producing inflammatory mediators upon capture and phagocytosis; second, through these preceding innate functions, they interpret the fungus-associated information and translate it into qualitatively different T-helper (Th) responses, and third they have a key role in containing and dampening inflammatory responses by tolerization through the induction of T-cells, thereby bridging the innate and adaptive immune systems [65], [66].

After contact with *A. fumigatus*, iDCs undergo maturation responsible for an enhanced expression of cytokines, chemokines, co-stimulatory molecules and an improved antigen presentation for efficient stimulation of naive T cells [67]. Our data demonstrate that various cytokines and chemokines were up-regulated during co-cultivation with *A. fumigatus*, including *CCL4*, *CCL5*, and *CXCL1–CXCL3*, resulting in activation and mobilization of other first-line immune effector cell populations, including neutrophils and macrophages. *CXCL2* (*MIP-2α*) showed the strongest up-regulation (19×) of any of the differentially regulated genes; independent of the fungal morphology present in the culture (from conidia to germ tubes to hyphae). It has been shown that CXCL2 is an extremely active chemotactic protein, mainly to PMNs, but induces limited chemokinetic activity. It can also induce degranulation of PMN, underlining the interaction between activated iDCs and PMNs. In parallel, we observed a strong up-regulation of *CCL4* (3 h–12 h, approximately 11×), which is chemoattractive for NK cells and *CCL5* (also known as RANTES), which plays an active role in recruiting T-lymphocytes into inflammatory sites. Thus, it can be hypothesized that CXCL2, CCL4 and CCL5 (together with other cytokines) in iDCs orchestrate the activation of the innate and adaptive immune system for effective management of *A. fumigatus* infection [68].

Immune gene regulation in iDCs also appeared to be responding to specific fungal stimuli. It has been reported that IL8 is expressed in response to germ tubes of *A. fumigatus* [29] and the increase in IL8 production after 3 h in this study may have been due to germ tube emergence (Table 1). Furthermore, there was up-regulation of *CCL20* (at 6 h–12 h of co-cultivation). Interestingly, we previously demonstrated that the *A. fumigatus* antigen Aspf1 is able to induce expression of *CCL20* [33]. In this recent study, we were able to show that in parallel to the augmented expression of *CCL20* in iDCs, *Aspf1* was up-regulated at the same time points in the fungus.

It is also worth noting that the gene encoding the IkappaB inhibitor was up-regulated during infection with *A. fumigatus*, despite of the fact that *NFkappaB* itself was not markedly differentially regulated. NF-kappaB activity is regulated by cytoplasmic degradation of the IkappaB inhibitor and nuclear translocation. When cells are stimulated, *e. g.* by *A. fumigatus*, the IkappaB inhibitor is rapidly phosphorylated and then degraded by proteasomes, allowing translocation of NFkappaB to the nucleus [69]. It could be speculated that increased expression of the IkappaB inhibitor gene in iDCs prevents uncontrolled release of inflammatory mediators (hypercytokinemia), which is a potentially fatal immune reaction.

A delayed response to *A. fumigatus* (Fig. 4) could lead to increased susceptibility to aspergillosis; the significance of donor variability has been confirmed by the association of SNP in CXCL10 and an increased risk of IA [70].

This parallel analysis of the transcriptomes of *A. fumigatus* and human iDCs revealed changes in gene expression that occurred in both organisms when they interact from the point of contact up to 12 hours later, which is likely to represent a critical time period in the pathogen-host interaction and pathogen clearance. The pattern of fungal response is consistent with the nutritional and environmental stresses associated with its developing from conidia to germ tubes from within the phagosome of iDCs. There was less emphasis on virulence (or pathogenicity) related gene expression. The gene expression response of the iDCs was consistent with a pro-inflammatory response including evidence of a response to the developing fungus, *e.g.* the up-regulation of *CCL20* in iDCs mirrored the up-regulation of *Aspf1* in the fungus. In conclusion these data indicate that interaction analyses of human primary immune cells with *A. fumigatus* supplement existing results from animal models. Further studies are highly warranted to obtain a more precise understanding of the pathogenicity of *A. fumigatus*, its response to challenge by the host immune system and understanding human innate immunity to *Aspergilli*. Future studies have the potential to reveal new tools for improved diagnosis and alternative treatment options for this devastating disease.

## Materials and Methods

### Ethics Statement

This study, using whole blood specimens obtained from human healthy volunteer donors, was approved by the Ethical Committee of the University Hospital of Wuerzburg. Informed consent was written and provided by all study participants. Data analysis was conducted anonymously.

### Isolation of PBMC and generation of dendritic cells

Peripheral blood monocytes (PBMCs) were purified from donor buffy coat using MACS (Miltenyi) CD14 positive selection and induced to become immature dendritic cells by incubation with IL-4 and GM-CSF [13]. Human immature dendritic cells were generated from three different donors.

### Preparation of conidia

*Aspergillus fumigatus* (strain Af293) was grown on malt extract agar until the mycelium was covered in conidia. The conidia were purified by adding 3 ml sterile distilled water (SDW) to the surface of the plate and using a cotton swab to disturb the surface of the mycelium. The solution of conidia was passed through a 40 µm cell strainer (Becton Dickinson) and the concentration of conidia was determined using a haemocytometer.

### Co- culture of *A. fumigatus* and iDC

Human monocyte-derived immature dendritic cells (iDC), 6×10^5^ ml^−1^, were co-incubated with *A. fumigatus*, 3×10^6^ ml^−1^, in 1 ml RPMI (10% FCS); MOI of 5. *A. fumigatus* was grown at a concentration of 3×10^6^ ml^−1^, in 1 ml RPMI (10% FCS) as a control. The samples were incubated at 37°C and 5% CO_2_ in 1.5 ml microcentrifuge tubes for 0, 3, 6, 9 and 12 h; there were three replicates, each using dendritic cells from a different donor.

### Extraction of RNA

RNA was extracted from *A. fumigatus*, with or without iDC, immediately at each time point with the Ribo-Pure Yeast kit (Ambion). The co-culture was centrifuged at 10000 *g* for 3 min, and the supernatant taken off. The samples were washed twice in ice-cold PBST (PBS pH 7.4, 0.05% Tween) and vortex mixed for 10 s to lyse the dendritic cells. The samples were centrifuged, transferred to screw cap tubes containing ceramic beads and resuspended in lysis buffer, SDS and phenol∶chloroform (reagents provided with the kit). The samples were disrupted by 4×1 min cycles in a MagnaLyser (Roche) with 1 min on ice between cycles. The manufacturer's instructions were followed thereafter. The extracted RNA was stored at −80°C.

Total RNA was extracted from dendritic cells immediately at each time point, with or without *A. fumigatus*, using the RNeasy mini kit and QiaShredder spin columns (Qiagen) as per the manufacturer's instructions. RNA was eluted in 35 µl of RNase-free water and the concentration was quantified with the Nanodrop spectrophotometer (Peqlab).

### Fungal interaction with immature dendritic cells

Samples (50 µl) from the co-cultures of *A. fumigatus* and iDCs were taken and incubated with neutral red [23] in a 96 well plate. Since neutral red has been used as a viability indicator for mammalian cells it was possible to use it for both cell types present in the co-culture [71]. These were observed by light microscopy to determine the numbers of viable cells and measure the number of conidia directly interacting with iDCs. Conidia that were in direct contact with the boundary or within the boundary of iDC were termed iDC associated since absolute confirmation of phagocytosis was not possible using the methodology of this study. Cells that took up dye and compartmentalised it were scored as viable whereas cells that were homogenously stained or unstained for iDCs were scored as dead. Cells/conidia were expressed as a percentage based on the ratio of dead cells/ viable cells observed per treatment from a minimum of 200 cells per replicate. The development of fungal morphotypes was also monitored by light microscopy. Morphotypes were defined as resting conidia, swollen conidia (larger than resting conidia and with thinner walls due to the absence of the rodlet layer), germ tubes (the first emerging hyphal tip from the conidium that can still be phagocytosed), hyphae (longer than germ tubes, >50 µm) [72].

### Transcription profiling of *A. fumigatus* with a whole genome microarray

To examine the effect of iDC on *A. fumigatus* gene expression RNA was extracted from *A. fumigatus* grown in RPMI medium with or without iDC. *A. fumigatus* plus iDC was compared to *A. fumigatus* minus iDC at each time point; with *A. fumigatus* minus iDC acting as the reference condition. Transcriptional profiling in this study was achieved using the *A. fumigatus* (Af293) DNA amplicon array containing 9516 genes [73]. Each gene was present in triplicate on the array and each sample was hybridized with a flip-dye replicate in order to account for labelling bias. Microarrays were performed as described by JCVI SOPs m007 and m008 available at http://pfgrc.jcvi.org/index.php/microarray/protocols.html, two independent biological replicates were hybridised and analysed. Briefly, cDNA was generated from 2 µg of total RNA by incubating for 18 h at 42°C with Superscript II reverse transcriptase (Invitrogen), a dNTP mixture containing amino-allyl dTTP and primed with random hexamers. Unincorporated dNTPs and hexamers were removed by purification with a Qiagen PCR purification column. Samples were then incubated for 18 h at room temperature with either Cy-3 or Cy-5 dye. After labelling, unincorporated dye was removed by purification with a Qiagen PCR purification column. Dye-coupled cDNA probes were evaporated and resuspended in hybridization buffer (40% formamide, 5× SSC, 0.1% SDS, 0.1 mM DTT and 6% Salmon sperm DNA). The probe was heated for two cycles of 5 min at 95°C and then applied to the slide and incubated at 42°C for 18 h. After post-incubation washes (two cycles of 55°C in 2× SSC, 0.1% SDS; RT in 0.1× SSC, 0.1% SDS and RT 0.1× SSC), slides were scanned with a Genepix 6000B scanner to generate images that were analyzed with the Spotfinder program [74]. Exported fluorescence intensities were normalized in MIDAS [74] using LOWESS (Locally Weighted Scatterplot Smoothing), followed by flip-dye consistency check and in-slide-replicate analysis.

Genes showing differential expression across the time points of the experiment were identified using SAM (multi-class) analysis [75] implemented in the MEV program of the TM4 microarray data analysis suite (http://TM4.org). The delta value cut-off was chosen as the value that captured the maximum number of significant genes while maintaining the estimated false discovery rate at zero; the log_2_ cut-off was zero to allow identification of all genes showing differential expression across the time points. The genes identified by SAM were organised by the similarity of their expression patterns by hierarchical clustering using Euclidean distance and the average linkage clustering method of MEV. Gene ontology was determined using EASE (Expression Analysis Systematic Explorer) in TM4 to identify overrepresented Gene Ontology terms in the dataset, p-values are Fisher's exact probabilities that the terms were more highly represented in the dataset. FunCat analysis was done using the online interface at http://www.omnifung.hki-jena.de/csp/protecs/menu.csp; the p-value generated by Fisher's exact test indicated the significance of the number of hits for each category in the dataset taking the number of hits for the whole genome as a reference.

### RT-qPCR Analysis of differentially regulated *A. fumigatus* genes

cDNA were generated from 1 µg of total RNA using the Taqman reverse transcription kit (Applied Biosystems) according to manufacturer's instructions in a total volume of 100 µl.

20 µl qPCR reactions were set up using 1 µl of cDNA, 10 µl of 2× SYBR Green qPCR master mix (Applied Biosystems), 2 µl of 5M betaine (Sigma) and primers at a final concentration of 0.2 µM. RT-qPCR reactions were performed in 96 well plates in an ABI PRISM 7900HT Fast Real Time thermocycler. The program used consisted of an initial 10 min incubation at 95°C, followed by 40 cycles of 15 s at 95°C and 1 min at 62°C. Primers (Table S5) were designed using Primer 3 Plus [76] and specificity was verified by use of a melt curve step following the last amplification cycle.

C_T_ values were collected with a manual threshold of 0.2. Each target was tested in triplicate and the average of the three C_T_ values was used for analysis. The average C_T_ was converted to an approximate transcript number by the equation n = 2^(40−Ct)^. These values were then standardized to the determined value for *actA* and were reported as number of target transcripts per *actA* transcript. The values for the treatment and control samples were used to produce Log_2_ ratio values to compare to the microarray results.

### Analysis of iDC surface marker expression

Immature DCs in RPMI medium containing 10% FCS were seeded in a 24-well plate (2×10^5^ per well), followed by addition of *A. fumigatus* conidia (MOI = 5). As controls, cells were either treated with 1µg ml^−1^ LPS (Sigma) or left untreated. At defined time points (0 h, 3 h, 6 h, 9 h, 12 h) cells were collected and washed with 500 µl HBSS buffer containing 10% FCS (2000 rpm, 2 min at RT). After washing, samples were resuspended in 200 µl of HBSS (containing 10% FCS). Then PE-conjugated CD80 (BD), CD86 (BD), CD83 (BD) and CD40 (Immunotec) antibodies were added, and cells were incubated for 30 min on ice, followed by centrifugation at 2000 rpm for 2 min at RT and resuspension in 200 µl of HBSS (containing 10% FCS). Flow cytometric analysis was performed with a FACScalibur cytometer (BD) and the CellQuest software (BD).

### Transcription analysis of iDC by immune array

#### Spotting of custom-made arrays

Genes were chosen for being differentially regulated in genome-wide expression profiling studies in co-cultures of *A. fumigatus* with iDCs [13] or with PMNs (Loeffler *et al.*, 2009, *35^th^ EBMT*) or to be generally relevant for regulation of immune defence mechanisms. 117 oligos were designed by an algorithm of Operon with a length of 55 to 70 nucleotides and oligos were spotted onto glass slides using an automatic spotter (Genpak).

### Microarray-analysis protocol for iDC

400 ng of total RNA was amplified using the “MessageAmp™ II aRNA Amplification Kit” from Ambion. In general, 50 µg aRNA was obtained and aRNA of single samples (stimulated and un-stimulated) was Cy3-labelled and a pool of all samples was Cy5-labelled by *in vitro* transcription from 500 ng of aRNA, respectively (LabelStar Array Kit, Qiagen). 20 µl of a mixture of Cy3- and Cy5- labelled cDNA was resuspended in 480 µl Nexterion Oligo Hyb Buffer (Peqlab); heated to 95°C for 3 min and incubated for 16 h at 45°C on the custom-made arrays. The arrays were washed and scanned by using a ScanArray 4000 (Perkin Elmer).

For evaluation and analysis of array data, software packages from the Bioconductor project (www.bioconductor.org; [77]) were run under R (www.r-project.org). Data was normalised by variance stabilizing normalization (VSN; [78]) and differentially expressed genes were detected using the ‘Limma’ (Linear Models for Microarray Analysis) package [79]; implementing the empirical Bayes linear modelling approach [80]. A gene was considered to be significantly differentially regulated, if the p-value<0.05 and log_2_ fold change >1.

### RT-qPCR validation of human immune array

Human RNA (500 ng) was reverse transcribed with the QuantiTect RT kit (Qiagen), containing a blend of oligo dT and random primers. RT-qPCR assays were performed using a StepOnePlus instrument (Applied Biosystems) using the TaqMan Universal PCR mix (Applied Biosystems) and commercially available primers and probes (Gene Expression assays) for *TNF-α*, *IL-1ß*, *IL-8* and *CCL4*. All RT-PCR assays were run with an initial denaturation step (10 min at 95°C), followed by 40 cycles of repeated denaturation (15 s at 95°C) and primer annealing and extension (60 s at 60°C).

### Microarray accession numbers

The microarray data for iDC gene expression were submitted to the Gene Expression Omnibus (GEO, NCBI) under the accession numbers GSE21353, GPL10270. The *A. fumigatus* gene expression data were submitted to GEO and has the accession number GSE22053; the A. *fumigatus* custom PCR amplicon microarray is listed with the GEO accession number GPL10341.

## Supporting Information

Figure S1 **Chromosomal locations of each gene differentially regulated by *A. fumigatus* interacting with iDC over 12 h.** The numbering scheme for physically linked, co-regulated gene clusters was taken from McDonagh *et al.* [14].
(TIF)

Table S1 **Genes differentially expressed by *A. fumigatus* during a 12 h co-incubation with human immature dendritic cells.** Genes were identified by SAM (multi-class) analysis of whole genome transcription at 0 h, 3 h, 6 h, 9 h, 12 h.
(DOC)

Table S2 **Gene Ontology analysis of biological processes up-regulated by *A. fumigatus* during infection of iDC^1^.**
(DOC)

Table S3 **Gene Ontology analysis of biological processes down-regulated by *A. fumigatus* during infection of iDC^1^.**
(DOC)

Table S4 **Differentially expressed *A. fumigatus* genes located in the subtelomeric regions of each chromosome during infection of iDC.**
(DOC)

Table S5 **Oligonucleotides used for quantitative real time PCR analysis of *A. fumigatus* gene expression.**
(DOC)
