# Supplementary figures and images for: The Temporal Dynamics of Differential Gene Expression in Aspergillus fumigatus Interacting with Human Immature Dendritic Cells In Vitro
Source: PLoS One. 2011 Jan 14;6(1):e16016. doi: 10.1371/journal.pone.0016016 (PMC3021540; doi:10.1371/journal.pone.0016016)

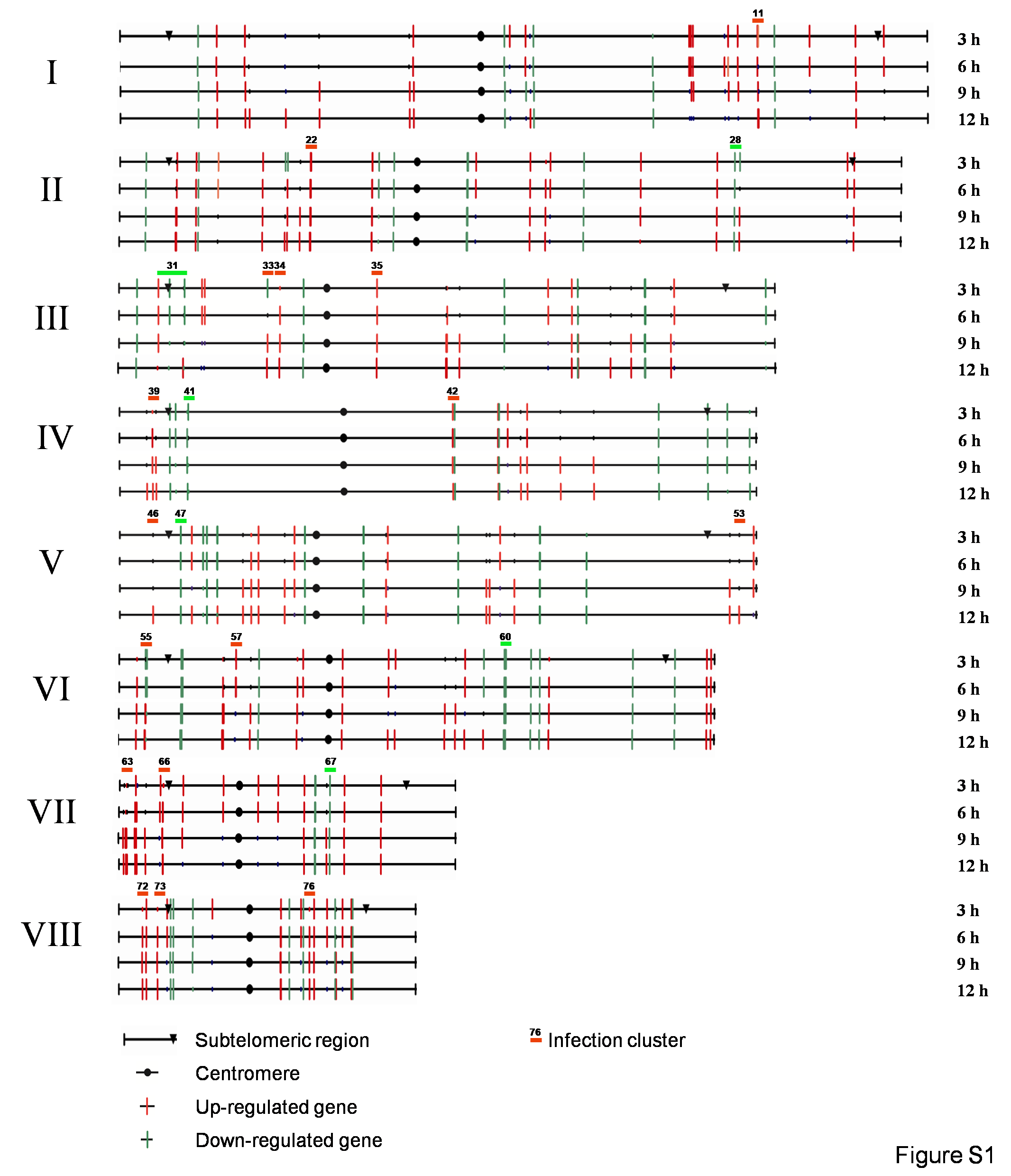

Supplement: Figure S1 — Chromosomal locations of each gene differentially regulated by A. fumigatus interacting with iDC over 12 h. The numbering scheme for physically linked, co-regulated gene clusters was taken from McDonagh et al. [14]. (TIF) [file pone.0016016.s001.tif]
