# Supplementary material for: The Temporal Dynamics of Differential Gene Expression in Aspergillus fumigatus Interacting with Human Immature Dendritic Cells In Vitro
Source: PLoS One. 2011 Jan 14;6(1):e16016. doi: 10.1371/journal.pone.0016016 (PMC3021540; doi:10.1371/journal.pone.0016016)
Supplement: Table S1 — Genes differentially expressed by A. fumigatus during a 12 h co-incubation with human immature dendritic cells. Genes were identified by SAM (multi-class) analysis of whole genome transcription at 0 h, 3 h, 6 h, 9 h, 12 h. (DOC) [file pone.0016016.s002.doc]

**Table S1** Genes differentially expressed by *A. fumigatus* during a 12 h co-incubation with human immature dendritic cells**.** Genes were identified by SAM (multi-class) analysis of whole genome transcription at 0h, 3h, 6h, 9h, 12h.

| **ORF** | **FUNCAT ID** | **Name** | **Log 2 relative fold change** | | | | | | | | | |
| --- | --- | --- | --- | --- | --- | --- | --- | --- | --- | --- | --- | --- |
|  |  |  | **Af 0h 1** | **Af 0h 2** | **Af 3h 1** | **Af 3h 2** | **Af 6h 1** | **Af 6h 2** | **Af 9h 1** | **Af 9h 2** | **Af 12h 1** | **Af 12h 2** |
| **Class I** | | | | | | | | | | | | |
| Afu2g03860 | AFUA_2g03860 | plasma membrane low affinity zinc ion transporter, putative | 0.64 | -1.15 | 3.19 | 2.62 | 3.03 | 2.49 | 2.97 | 2.80 | 3.01 | 2.69 |
| Afu4g09580 | AFUA_4g09580 | major allergen Asp F2 | 0.70 | -0.82 | 3.75 | 3.49 | 3.46 | 3.17 | 3.77 | 2.71 | 3.97 | 3.21 |
| Afu7g00340 | AFUA_7g00340 | metallo-beta-lactamase domain protein, putative | 0.53 | -0.16 | 1.78 | 2.41 | 2.29 | 3.04 | 2.72 | 3.11 | 2.46 | 3.23 |
| Afu3g03640 | AFUA_3g03640 | MFS siderochrome iron transporter MirB | NaN | -0.09 | 0.09 | 0.28 | 2.37 | 1.26 | 2.16 | 3.31 | 3.62 | 2.10 |
| Afu7g01000 | AFUA_7g01000 | potassium-activated aldehyde dehydrogenase Ald4, putative | 0.00 | -0.01 | 0.79 | 0.43 | 2.85 | 2.82 | 3.91 | 3.66 | 3.70 | 3.11 |
| Afu8g00610 | AFUA_8g00610 | cell surface protein Mas1, putative | NaN | 0.06 | -0.23 | -0.28 | 2.27 | 2.38 | 3.68 | 3.46 | 2.81 | 2.84 |
| Afu6g00430 | AFUA_6g00430 | IgE-binding protein | NaN | -0.24 | -0.84 | -0.46 | 1.22 | 1.09 | 2.49 | 2.19 | 2.04 | 2.17 |
| Afu7g00170 | AFUA_7g00170 | dimethylallyl tryptophan synthase GliD1 | 0.34 | -0.42 | -0.36 | -0.56 | 0.72 | 0.52 | 1.91 | 1.82 | 1.73 | 1.81 |
| Afu4g00750 | AFUA_4g00750 | conserved hypothetical protein | NaN | -0.25 | -0.19 | -0.24 | 1.30 | 0.93 | 2.24 | 1.30 | 2.97 | 2.39 |
| Afu5g03010 | AFUA_5g03010 | conserved hypothetical protein | -0.25 | -0.34 | 0.56 | 0.62 | 0.80 | NaN | 1.58 | 1.50 | 1.77 | 1.74 |
| Afu6g04680 | AFUA_6g04680 | MIPC synthase subunit (SurA), putative | -0.81 | 0.07 | 0.66 | 0.55 | 0.99 | 1.05 | 1.40 | 1.34 | 1.74 | 1.77 |
| Afu8g01030 | AFUA_8g01030 | conserved hypothetical protein | NaN | -0.26 | 0.27 | 0.19 | 1.03 | 0.85 | 1.42 | 1.18 | 1.88 | 1.60 |
| Afu8g04370 | AFUA_8g04370 | GPI anchored protein, putative | -0.43 | -0.11 | 0.57 | 0.21 | 1.43 | 0.97 | 1.49 | 1.54 | 1.84 | 1.83 |
| Afu3g07910 | AFUA_3g07910 | UDP-glucose 4-epimerase, putative | NaN | 0.10 | 0.26 | -0.09 | 1.11 | 0.88 | 1.75 | 1.64 | 2.07 | 1.94 |
| Afu6g10660 | AFUA_6g10660 | ATP citrate lyase subunit (Acl), putatibe | -0.09 | 0.25 | 0.37 | -0.21 | 1.09 | 0.61 | 1.40 | 1.13 | 1.93 | 1.67 |
| Afu6g03050 | AFUA_6g03050 | oleate delta-12 desaturase | -0.20 | 0.15 | 0.41 | 0.25 | 1.19 | 1.21 | 1.62 | 1.57 | 2.43 | 2.53 |
| Afu2g04200 | AFUA_2g04200 | 4-hydroxyphenylpyruvate dioxygenase, putative | -0.28 | -0.14 | 0.99 | 1.06 | 0.96 | 1.23 | 1.43 | 1.88 | 1.71 | 2.66 |
| Afu8g05950 | AFUA_8g05950 | conserved hypothetical protein | -0.82 | -0.05 | 1.07 | 0.80 | 1.33 | 1.28 | 1.59 | 1.72 | 1.45 | 1.99 |
| Afu5g14430 | AFUA_5g14430 |  | NaN | -0.11 | 0.82 | 0.71 | 1.44 | 0.95 | 1.78 | 1.89 | 1.38 | 1.91 |
| Afu2g10230 | AFUA_2g10230 | inositol oxygenase, putative | -0.07 | 0.02 | 0.71 | 0.58 | 1.36 | 1.56 | 1.60 | 1.60 | 1.46 | 2.25 |
| Afu8g05010 | AFUA_8g05010 | C2H2 finger domain protein, putative | -0.48 | -0.54 | 0.66 | 0.31 | 1.56 | 1.18 | 1.90 | 1.31 | 1.69 | 2.24 |
| Afu7g06540 | AFUA_7g06540 | threonine aldolase, putative | -0.01 | -0.13 | 2.27 | 2.70 | 2.16 | 2.72 | 1.79 | 1.77 | 1.51 | 2.16 |
| Afu7g00990 | AFUA_7g00990 | transcriptional activator of ethanol catabolism AlcS | 0.44 | NaN | NaN | 0.37 | 1.65 | 1.89 | 2.25 | 2.30 | 1.25 | 1.71 |
| Afu2g16860 | AFUA_2g16860 | MFS multidrug transporter, putative | -0.54 | -0.34 | 1.66 | 1.32 | 2.28 | 1.91 | 2.59 | 2.19 | 1.72 | 1.82 |
| Afu7g04820 | AFUA_7g04820 | C6 transcription factor, putative | 0.09 | -0.49 | 1.27 | 1.51 | 1.35 | 1.77 | 2.08 | 2.25 | 1.97 | 2.18 |
| Afu8g04380 | AFUA_8g04380 | conserved hypothetical protein | 0.44 | -0.18 | 1.46 | 1.60 | 1.67 | 2.05 | 2.06 | 2.17 | 1.82 | 2.03 |
| Afu8g05090 | AFUA_8g05090 | MFS transporter, putative | -0.24 | -0.04 | 1.32 | 1.27 | 1.59 | 2.01 | 1.90 | 2.24 | 1.77 | 2.49 |
| Afu1g06190 | AFUA_1g06190 | histone H4 arginine methyltransferase RmtA | -0.64 | 0.02 | 1.69 | 1.05 | 1.85 | 1.34 | 1.42 | 1.62 | 1.68 | 2.19 |
| Afu2g09860 | AFUA_2g09860 | purine-cytosine permease | -0.73 | 0.01 | 1.42 | 1.17 | 1.41 | 1.38 | 1.39 | 1.39 | 1.15 | 1.60 |
| Afu3g10660 | AFUA_3g10660 | hydroxymethylglutaryl-CoA synthase Erg13, putative | -0.76 | 0.00 | 1.32 | 0.91 | 1.69 | 1.28 | 1.56 | 1.35 | 1.46 | 1.55 |
| Afu2g05550 | AFUA_2g05550 | conserved hypothetical protein | 0.22 | 0.31 | 1.11 | 1.17 | 1.49 | 1.56 | 1.34 | 1.22 | 1.18 | 1.08 |
| Afu6g14260 | AFUA_6g14260 | U3 small nucleolar ribonucleoprotein protein Lcp5, putative | 0.19 | 0.17 | 1.29 | 1.23 | 1.53 | 1.52 | 1.20 | 1.28 | 1.01 | 1.00 |
| Afu1g02610 | AFUA_1g02610 | rRNA processing protein, putative | 0.04 | 0.22 | 1.10 | 1.19 | 1.36 | 1.38 | 1.03 | 1.15 | 1.00 | 1.15 |
| Afu2g01480 | AFUA_2g01480 | 60S ribosomal subunit biogenesis protein Nop16, putative | 0.29 | 0.22 | 1.03 | 1.27 | 1.39 | 1.55 | 1.25 | 1.25 | 1.55 | 1.43 |
| Afu1g01990 | AFUA_1g01990 | ribosome biogenesis protein Rsa4, putative | -0.31 | -0.23 | 1.25 | 1.25 | 1.44 | 1.41 | 1.25 | 1.16 | 1.37 | 1.31 |
| Afu2g05560 | AFUA_2g05560 | exonuclease, putative | 0.03 | 0.07 | 1.15 | 1.28 | 1.28 | 1.31 | 1.24 | 1.30 | 1.21 | 1.41 |
| Afu5g03140 | AFUA_5g03140 | GTP cyclohydrolase I, putative | -0.17 | 0.08 | 1.25 | 1.02 | 1.42 | 1.18 | 1.14 | 1.30 | 1.25 | 1.60 |
| Afu8g00720 | AFUA_8g00720 | amino acid transporter, putative | -0.33 | -0.09 | 0.99 | 1.20 | 1.40 | 1.46 | 1.15 | 1.20 | 0.81 | 1.12 |
| Afu3g06380 | AFUA_3g06380 | exosome-associated family protein | -0.25 | -0.12 | 1.25 | 1.31 | 1.31 | 1.30 | 1.15 | 0.89 | 0.95 | 1.31 |
| Afu4g08930 | AFUA_4g08930 | nucleolar GTPase, putative | -0.50 | -0.14 | 1.15 | 0.76 | 1.34 | 1.04 | 1.07 | 1.05 | 1.00 | 1.41 |
| Afu2g03340 | AFUA_2g03340 | DUF652 domain protein | -0.37 | -0.03 | 1.37 | 1.03 | 1.44 | 1.26 | 0.99 | 1.18 | 0.92 | 1.37 |
| Afu4g07810 | AFUA_4g07810 | L-serine dehydratase, putative | 0.19 | -0.22 | 1.88 | 1.48 | 1.30 | 1.57 | 1.02 | 1.12 | 1.07 | 1.41 |
| Afu1g16550 | AFUA_1g16550 | dihydrouridine synthase family protein, putative | 0.04 | -0.20 | 1.89 | 1.74 | 1.59 | 1.48 | 1.07 | 1.02 | 0.92 | 1.11 |
| Afu7g06530 | AFUA_7g06530 | hypothetical protein | 0.10 | -0.13 | 1.69 | 1.95 | 1.28 | 1.51 | 0.92 | 0.90 | NaN | 0.80 |
| Afu3g01030 | AFUA_3g01030 | RTA1 domain protein, putative | 0.14 | -0.20 | 1.83 | 1.76 | 1.27 | 1.46 | 0.94 | 1.01 | 0.64 | 0.99 |
| Afu2g12450 | AFUA_2g12450 | hydroxymethylglutaryl-CoA lyase | -0.01 | -0.06 | 1.75 | 1.82 | 1.31 | 1.45 | 1.05 | 1.10 | 0.61 | 1.02 |
| Afu7g05650 | AFUA_7g05650 | XYPPX repeat family protein | -0.04 | -0.20 | 1.70 | 1.92 | 1.29 | 1.13 | 1.11 | 1.00 | 1.31 | 1.37 |
| Afu1g14540 | AFUA_1g14540 | oxidoreductase, short-chain dehydrogenase/reductase family | 0.21 | -0.06 | 1.57 | 1.54 | 1.09 | 1.38 | 1.38 | 1.06 | 1.46 | 1.28 |
| Afu2g14020 | AFUA_2g14020 | ABC transporter, putative | 0.01 | 0.12 | 1.50 | 1.60 | 1.44 | 1.94 | 1.76 | 1.92 | 1.27 | 1.42 |
| Afu2g04230 | AFUA_2g04230 | fumarylacetoacetate hydrolase FahA | -0.03 | 0.09 | 1.67 | 1.69 | 1.39 | 1.80 | 1.51 | 1.70 | 1.31 | 2.09 |
| Afu2g01880 | AFUA_2g01880 | low molecular weight phosphotyrosine protein phosphatase, putative | -0.30 | -0.01 | 1.90 | 2.11 | 1.82 | 1.99 | 1.46 | 1.70 | 1.66 | 1.50 |
| Afu6g14510 | AFUA_6g14510 | monooxygenase, putative | -0.39 | 0.29 | 2.00 | 2.29 | 2.16 | 2.13 | 1.42 | 1.45 | 0.90 | 1.35 |
| **Class II** | | | | | | | | | | | | |
| Afu1g09650 | AFUA_1g09650 | GPI anchored protein, putative | -0.66 | -0.75 | -0.07 | 0.29 | 0.38 | 0.42 | 0.22 | 0.44 | 0.97 | 0.75 |
| Afu1g03490 | AFUA_1g03490 | epimerase/dehydratase family protein, putative | -0.84 | -0.73 | 0.58 | 0.52 | 0.41 | 0.41 | 0.56 | 0.67 | 0.81 | 1.18 |
| Afu6g07470 | AFUA_6g07470 | integral membrane protein | -0.50 | -0.51 | 0.79 | 0.74 | 0.50 | 0.53 | 0.60 | 0.64 | 1.17 | 1.73 |
| Afu7g00390 | AFUA_7g00390 | MFS multidrug transporter, putative | -0.71 | -0.66 | 0.09 | 0.40 | 0.46 | 0.93 | 0.77 | 1.00 | 0.63 | 1.18 |
| Afu7g02320 | AFUA_7g02320 | conserved hypothetical protein | -0.73 | -0.69 | 0.58 | 0.73 | 0.70 | 0.91 | 0.35 | 0.18 | 0.47 | 0.49 |
| Afu1g13800 | AFUA_1g13800 | MFS multidrug transporter, putative | -0.58 | -0.54 | 0.54 | 0.37 | 0.64 | 0.65 | 0.67 | 0.70 | 0.26 | 0.26 |
| Afu1g17060 | AFUA_1g17060 | HLH DNA binding domain protein, putative | 0.36 | -0.43 | 1.86 | 1.82 | 1.17 | 1.24 | 0.58 | 0.54 | 0.01 | 0.24 |
| Afu2g02390 | AFUA_2g02390 | conserved hypothetical protein | 0.22 | -0.11 | 1.51 | 1.79 | 1.23 | 1.20 | 0.78 | 0.67 | 0.36 | 0.50 |
| Afu5g06680 | AFUA_5g06680 | 4-aminobutyrate transaminase GatA | 0.02 | 0.27 | 1.70 | 1.77 | 0.68 | 0.81 | 0.16 | 0.00 | 0.24 | 0.10 |
| Afu3g02070 | AFUA_3g02070 | C2H2 transcription factor, putative | 0.23 | 0.19 | 1.69 | 1.38 | 1.27 | 0.81 | 0.56 | 0.36 | 0.16 | 0.03 |
| Afu1g15530 | AFUA_1g15530 | lactam utilization protein LamB | 0.47 | -0.01 | 1.54 | 1.48 | 0.95 | 0.91 | 0.60 | 0.32 | 0.13 | 0.04 |
| Afu6g03360 | AFUA_6g03360 | ToxD-like zinc binding oxidoreductase, putative | 0.16 | -0.32 | 1.80 | 1.41 | 0.65 | 0.75 | 0.60 | 0.41 | NaN | 0.19 |
| Afu7g03830 | AFUA_7g03830 | DNA repair protein Rad7, protein | -0.01 | 0.02 | 1.40 | 1.16 | 0.88 | 0.75 | 0.48 | 0.45 | 0.09 | 0.09 |
| Afu1g09570 | AFUA_1g09570 | conserved hypothetical protein | -0.07 | -0.15 | 1.25 | 1.22 | 0.83 | 0.91 | 0.52 | 0.46 | 0.34 | 0.07 |
| Afu3g12740 | AFUA_3g12740 | copper resistance-associated P-type ATPase, putative | -0.18 | -0.20 | 1.41 | 1.43 | 1.04 | 1.12 | 0.54 | 0.30 | 0.03 | 0.01 |
| Afu3g02110 | AFUA_3g02110 | MFS multidrug transporter, putative | -0.33 | -0.06 | 1.13 | 1.12 | 0.69 | 0.74 | 0.26 | 0.12 | NaN | -0.06 |
| Afu7g04290 | AFUA_7g04290 | amino acid permease (Gap1), putative | -0.57 | -0.23 | 1.51 | 1.48 | 1.12 | 1.00 | 0.85 | 0.70 | 0.17 | 0.46 |
| Afu5g15010 | AFUA_5g15010 | arsenite efflux transporter (ArsB), putative | -0.19 | -0.31 | 1.31 | 1.24 | 0.99 | 1.04 | 0.74 | 0.87 | 0.30 | 0.57 |
| Afu7g00930 | AFUA_7g00930 | integral membrane protein | -0.38 | -0.34 | 1.03 | 1.18 | 0.82 | 1.04 | 0.64 | 0.64 | 0.36 | 0.33 |
| Afu3g10150 | AFUA_3g10150 | conserved hypothetical protein | -0.84 | -0.56 | 1.20 | 0.85 | 1.09 | 1.04 | 0.59 | 0.62 | 0.31 | 0.57 |
| Afu6g03370 | AFUA_6g03370 | short-chain dehydrogenase/reductase, putative | -0.21 | -0.04 | 1.79 | 1.55 | 1.42 | 1.03 | 0.76 | 1.13 | 0.40 | 0.93 |
| Afu1g13110 | AFUA_1g13110 | 4-coumarate-CoA ligase, putative | -0.38 | -0.09 | 1.40 | 1.41 | 1.19 | 1.23 | 1.06 | 0.97 | 0.59 | 0.87 |
| Afu6g04820 | AFUA_6g04820 | para-aminobenzoate synthase PabaA | -0.40 | -0.03 | 1.67 | 1.48 | 1.14 | 1.16 | 0.94 | 1.02 | 0.70 | 0.93 |
| Afu1g13880 | AFUA_1g13880 | snoRNP assembly factor Naf1, putative | -0.42 | 0.02 | 1.38 | 1.09 | 1.45 | 1.23 | 0.85 | 1.00 | 0.51 | 0.79 |
| Afu5g09060 | AFUA_5g09060 | RNA binding protein, putative | -0.50 | -0.19 | 1.51 | 1.23 | 1.35 | 1.31 | 0.79 | 0.92 | 0.52 | 0.84 |
| Afu1g13160 | AFUA_1g13160 | geranylgeranyl diphosphate synthase | -0.07 | 0.04 | 1.26 | 1.35 | 1.11 | 1.41 | 0.96 | 1.06 | 0.69 | 0.66 |
| Afu2g16750 | AFUA_2g16750 | nonsense-mediated mRNA decay protein 3 | -0.13 | 0.11 | 1.34 | 1.13 | 1.52 | 1.38 | 1.03 | 0.99 | 0.83 | 0.86 |
| Afu1g14560 | AFUA_1g14560 | mannosidase MsdS | -0.26 | -0.35 | 1.02 | 0.82 | 0.92 | 0.65 | 0.89 | 0.92 | 0.98 | 1.04 |
| Afu6g07330 | AFUA_6g07330 | methionine aminopeptidase, type I, putative | -0.01 | -0.11 | 0.93 | 0.94 | 1.17 | 1.10 | 1.04 | 1.16 | 0.97 | 1.13 |
| Afu6g08910 | AFUA_6g08910 | tRNA methyltransferase, putative | -0.17 | -0.04 | 1.13 | 1.04 | 0.96 | 0.97 | 0.99 | 1.02 | 0.97 | 1.11 |
| Afu6g06380 | AFUA_6g06380 | methyltransferase, putative | -0.22 | -0.09 | 1.21 | 1.01 | 1.24 | 1.03 | 0.99 | 1.08 | 1.07 | 1.10 |
| Afu2g10330 | AFUA_2g10330 | conserved hypothetical protein | -0.15 | -0.11 | 1.22 | 1.11 | 1.22 | 1.00 | 1.07 | 1.17 | 0.90 | 0.79 |
| Afu8g01260 | AFUA_8g01260 | hypothetical protein | -0.23 | -0.22 | 0.99 | 0.93 | 0.98 | 1.12 | 0.89 | 0.87 | 0.56 | 0.68 |
| Afu5g04010 | AFUA_5g04010 | tRNA-splicing endonuclease subunit Sen2, putative | -0.40 | -0.28 | 0.93 | 0.89 | 1.13 | 0.99 | 0.97 | 0.90 | 0.50 | 0.58 |
| Afu8g05430 | AFUA_8g05430 | ribosome biogenesis protein Noc4, putative | -0.22 | -0.06 | 1.10 | 0.95 | 1.15 | 1.17 | 0.83 | 0.69 | 0.57 | 0.74 |
| Afu2g08480 | AFUA_2g08480 | ATP-dependent RNA helicase Mrh4, putative | -0.39 | -0.20 | 0.95 | 1.10 | 1.05 | 1.10 | 0.91 | 0.88 | 0.78 | 1.02 |
| Afu8g05710 | AFUA_8g05710 | MFS sugar transporter Stl1, putative | -0.22 | -0.30 | 1.18 | 1.11 | 1.21 | 1.14 | 0.96 | 0.68 | 0.78 | 1.10 |
| Afu7g01490 | AFUA_7g01490 | MFS peptide transporter Ptr2, putative | -0.77 | -0.12 | 1.29 | 1.19 | 1.14 | 1.31 | 1.26 | 1.15 | 1.04 | 1.32 |
| Afu5g01740 | AFUA_5g01740 | deoxyhypusine synthase, putative | -1.04 | -0.12 | 1.29 | 1.14 | 1.06 | 1.17 | 1.01 | 1.07 | 1.14 | 1.14 |
| Afu1g14080 | AFUA_1g14080 | integral membrane protein Pth11-like, putative | -0.73 | -0.57 | 1.02 | 0.95 | 1.37 | 1.18 | 1.09 | 0.96 | 0.56 | 0.97 |
| Afu4g09110 | AFUA_4g09110 | cytochrome c peroxidase Ccp1, putative | -0.58 | -0.02 | 1.26 | 1.18 | 1.07 | 1.04 | 0.79 | 0.76 | 0.69 | 0.94 |
| Afu8g04820 | AFUA_8g04820 | ribonuclease P complex subunit Pop2, putative | -0.77 | -0.27 | 1.40 | 1.22 | 1.07 | 1.10 | 0.68 | 0.57 | 0.79 | 0.91 |
| Afu1g13080 | AFUA_1g13080 | BRCT domain protein | -0.65 | -0.18 | 1.31 | 1.09 | 1.23 | 1.20 | 1.24 | 0.64 | NaN | 1.03 |
| Afu1g09140 | AFUA_1g09140 | conserved hypothetical protein | -1.43 | -0.75 | 1.31 | 1.05 | 0.72 | 0.69 | 0.26 | 0.31 | NaN | 0.62 |
| Afu8g02190 | AFUA_8g02190 | hypothetical protein | -0.81 | -0.28 | 1.56 | 1.22 | 0.62 | 0.61 | 0.10 | 0.27 | -0.05 | 0.23 |
| **Class III** | | | | | | | | | | | | |
| Afu5g14660 | AFUA_5g14660 | GABA permease, putative | -0.70 | -0.44 | 0.20 | 0.01 | -0.38 | -0.31 | 0.51 | 0.46 | 0.98 | 0.93 |
| Afu5g02330 | AFUA_5g02330 | major allergen and cytotoxin AspF1 | NaN | 0.07 | -0.77 | -0.68 | -0.87 | -0.61 | 0.10 | 0.15 | 1.02 | 1.16 |
| Afu6g09350 | AFUA_6g09350 | C6 finger domain protein, putative | 0.18 | 0.27 | -0.60 | -0.60 | -0.25 | -0.50 | 0.00 | 0.22 | 0.71 | 0.86 |
| Afu5g00720 | AFUA_5g00720 | GNAT family acetyltransferase, putative | NaN | NaN | -0.54 | -0.28 | -0.41 | -0.06 | 0.38 | 0.29 | 1.06 | 1.57 |
| Afu6g00620 | AFUA_6g00620 | GPI anchored hypothetical protein | -0.18 | -0.12 | -0.85 | -0.53 | -0.30 | -0.09 | 0.41 | 0.34 | 0.91 | 0.90 |
| Afu7g00580 | AFUA_7g00580 | conserved hypothetical protein | -0.05 | -0.18 | -0.24 | -0.34 | -0.17 | -0.22 | 0.60 | 0.57 | 0.65 | 0.77 |
| Afu6g00640 | AFUA_6g00640 | integral membrane protein | -0.20 | -0.27 | -0.30 | -0.30 | -0.54 | -0.41 | 0.55 | 0.52 | 1.05 | 0.97 |
| Afu6g00630 | AFUA_6g00630 | MFS transporter, putative | 0.30 | -0.21 | -0.62 | -0.22 | -0.60 | -0.47 | 0.82 | 0.70 | 1.53 | 1.08 |
| Afu3g01580 | AFUA_3g01580 | GMC oxidoreductase, putative | 0.14 | 0.03 | -0.05 | -0.04 | -0.54 | -0.48 | 0.35 | 0.24 | 1.60 | 1.24 |
| Afu4g00610 | AFUA_4g00610 | aryl-alcohol dehydrogenase, putative | 0.16 | -0.08 | -0.15 | 0.13 | -0.28 | -0.29 | 0.33 | 0.33 | 1.45 | 1.09 |
| Afu5g14330 | AFUA_5g14330 | 12-oxophytodienoate reductase, putative | NaN | NaN | -0.39 | 0.06 | -0.14 | -0.35 | 0.83 | 0.57 | 1.39 | 1.12 |
| Afu4g09390 | AFUA_4g09390 | DUF563 domain protein | 0.00 | 0.03 | -0.17 | -0.26 | 0.12 | -0.22 | 0.77 | 0.45 | 1.31 | 1.41 |
| Afu6g03680 | AFUA_6g03680 | hypothetical protein | NaN | 0.00 | -0.15 | 0.32 | NaN | 0.19 | 0.48 | 0.61 | 1.86 | 2.11 |
| Afu3g08110 | AFUA_3g08110 | cell wall protein, putative | NaN | -0.47 | 0.26 | 0.28 | 0.38 | 0.38 | 1.24 | 1.19 | 2.34 | 1.69 |
| Afu5g02850 | AFUA_5g02850 | conserved hypothetical protein | -0.12 | -0.13 | -0.08 | -0.22 | 0.02 | -0.14 | 1.19 | 1.21 | 2.24 | 1.69 |
| Afu6g03060 | AFUA_6g03060 | MFS monosaccharide transporter, putative | -0.25 | -0.52 | 0.01 | 0.21 | 0.94 | 1.00 | 1.25 | 0.92 | 1.18 | 1.15 |
| Afu2g04060 | AFUA_2g04060 | NADH:flavin oxidoreductase/NADH oxidase family protein | -0.57 | 0.00 | 0.56 | 0.70 | 0.87 | 1.10 | 1.36 | 1.18 | 1.34 | 1.24 |
| Afu6g08710 | AFUA_6g08710 | alkaline phosphatase, putative | -0.14 | -0.12 | 0.52 | 0.71 | 0.87 | 1.02 | 1.03 | 0.98 | 1.09 | 0.96 |
| Afu2g01440 | AFUA_2g01440 | mitochondrial carrier protein, putative | -0.34 | 0.17 | 0.21 | 0.25 | 1.00 | 0.90 | 1.17 | 1.06 | 1.35 | 1.31 |
| Afu1g04720 | AFUA_1g04720 | C-8 sterol isomerase (Erg-1), putative | -0.20 | -0.01 | 0.32 | 0.41 | 0.84 | 0.76 | 0.94 | 0.93 | 1.58 | 1.62 |
| Afu7g00120 | AFUA_7g00120 | metallo-beta-lactamase domain protein | 0.10 | -0.30 | 0.03 | 0.24 | 0.43 | 0.43 | 1.48 | 1.35 | 1.46 | 1.59 |
| Afu4g00830 | AFUA_4g00830 | MFS peptide transporter, putative | -0.05 | -0.14 | 0.00 | -0.09 | 0.47 | 0.55 | 1.21 | 1.16 | 1.24 | 1.29 |
| Afu5g03760 | AFUA_5g03760 | class III chitinase ChiA1 | -0.15 | -0.01 | 0.21 | 0.13 | 0.35 | 0.49 | 1.29 | 1.02 | 1.39 | 1.11 |
| Afu3g07890 | AFUA_3g07890 | endo alpha-1,4 polygalactosaminidase, putative | 0.00 | -0.09 | 0.18 | -0.11 | 0.77 | 0.49 | 1.42 | 1.14 | 1.48 | 1.44 |
| Afu5g08770 | AFUA_5g08770 | conserved hypothetical protein | 0.40 | 0.11 | 0.20 | 0.18 | 0.62 | 0.40 | 0.92 | 0.92 | 1.58 | 1.37 |
| Afu5g09400 | AFUA_5g09400 | carbonyl reductase, putative | 0.18 | -0.10 | -0.02 | 0.28 | 0.31 | 0.35 | 0.88 | 0.76 | 1.42 | 1.56 |
| Afu5g08830 | AFUA_5g08830 | Woronin body protein HexA, putative | -0.05 | 0.12 | -0.03 | 0.08 | 0.46 | 0.40 | 1.10 | 0.78 | 1.49 | 1.58 |
| Afu7g08330 | AFUA_7g08330 |  | -0.29 | -0.31 | 0.40 | 0.30 | 0.31 | 0.15 | 0.57 | 0.91 | 1.06 | 1.48 |
| Afu4g11130 | AFUA_4g11130 | conserved hypothetical protein | -0.20 | -0.37 | 0.23 | 0.26 | 0.44 | 0.39 | 0.91 | 0.79 | 1.29 | 1.67 |
| Afu4g10340 | AFUA_4g10340 | C6 finger domain protein, putative | -0.07 | -0.26 | 0.46 | 0.17 | 0.50 | 0.03 | 0.86 | 0.71 | 1.41 | 1.39 |
| Afu7g05300 | AFUA_7g05300 | conserved hypothetical protein | NaN | NaN | 0.11 | -0.08 | 0.77 | 0.55 | 1.01 | 0.95 | 0.99 | 0.91 |
| Afu3g10770 | AFUA_3g10770 | RTA1 domain protein, putative | -0.29 | -0.25 | -0.27 | -0.03 | 0.57 | 0.64 | 0.94 | 0.88 | 0.84 | 0.82 |
| Afu3g11430 | AFUA_3g11430 | arginase, putative | -0.29 | -0.21 | -0.15 | -0.04 | 0.57 | 0.25 | 0.63 | 0.56 | 1.05 | 0.98 |
| Afu1g06090 | AFUA_1g06090 | conserved hypothetical protein | -0.29 | -0.51 | 0.04 | -0.03 | 0.47 | 0.31 | 0.54 | 0.76 | 0.96 | 1.04 |
| Afu6g08510 | AFUA_6g08510 | cell wall glucanase, putative | NaN | NaN | 0.07 | -0.22 | 0.48 | 0.09 | 0.88 | 0.81 | 1.64 | 1.35 |
| Afu3g11860 | AFUA_3g11860 | microtubule associated protein EB1, putative | -0.61 | 0.11 | 0.20 | 0.00 | 0.67 | 0.28 | 1.00 | 0.73 | 1.49 | 1.63 |
| Afu5g06660 | AFUA_5g06660 | UPF0187 domain membrane protein | NaN | -0.17 | -0.26 | -0.22 | 0.55 | 0.51 | 0.97 | 0.58 | 1.61 | 1.23 |
| Afu3g12690 | AFUA_3g12690 | conserved hypothetical protein | -0.52 | -0.34 | 0.24 | -0.15 | 0.76 | 0.65 | 0.94 | 0.70 | 1.31 | 1.43 |
| Afu2g14540 | AFUA_2g14540 | endoglucanase, putative | NaN | NaN | -0.92 | -0.75 | 0.64 | 0.73 | 1.22 | 0.77 | 1.28 | 0.88 |
| Afu3g03410 | AFUA_3g03410 | enoyl-CoA hydratase/isomerase family protein | NaN | -0.05 | -0.65 | -0.50 | 0.53 | 0.53 | 1.35 | 0.86 | 1.02 | 0.47 |
| Afu7g00200 | AFUA_7g00200 | conserved hypothetical protein | NaN | -0.13 | -0.52 | -0.16 | 0.28 | 0.09 | 1.27 | 1.05 | 1.22 | 1.00 |
| Afu2g03830 | AFUA_2g03830 | allergen Asp F4 | NaN | NaN | -0.54 | -0.81 | 0.29 | -0.06 | 0.74 | 0.45 | 1.11 | 1.08 |
| Afu8g05610 | AFUA_8g05610 | cell wall glucanase (Scw11), putative | NaN | -0.22 | -0.29 | -0.48 | 0.26 | 0.12 | 0.98 | 0.60 | 1.43 | 0.97 |
| Afu7g04790 | AFUA_7g04790 | conserved hypothetical protein | -0.35 | -0.15 | 0.06 | -0.34 | 0.15 | 0.04 | 0.72 | 0.53 | 1.04 | 1.30 |
| Afu1g02730 | AFUA_1g02730 | mitochondrial phosphate carrier protein (Ptp), putative | -0.43 | 0.06 | -0.17 | -0.11 | 0.32 | 0.33 | 0.65 | 0.62 | 1.20 | 1.45 |
| **Class IV** | | | | | | | | | | | | |
| Afu4g01580 | AFUA_4g01580 | ankyrin repeat protein | NaN | -0.15 | -1.31 | -1.30 | 0.23 | 0.13 | 0.37 | 0.31 | NaN | -0.12 |
| Afu6g09710 | AFUA_6g09710 | MFS gliotoxin efflux transporter GliA | -0.45 | -0.41 | -1.14 | -1.09 | -2.16 | -1.63 | -2.16 | -2.04 | -1.71 | -2.25 |
| Afu6g09680 | AFUA_6g09680 | O-methyltransferase GliM | 0.58 | -0.16 | -1.35 | -0.86 | -2.43 | -2.24 | -2.42 | -2.37 | -1.96 | -2.55 |
| Afu2g11120 | AFUA_2g11120 | conserved hypothetical protein | 0.80 | -0.35 | -2.02 | -2.41 | -2.67 | -2.73 | -2.48 | -2.48 | -1.88 | -2.56 |
| Afu3g12190 | AFUA_3g12190 | RING finger domain protein, putative | 0.16 | 0.31 | -1.95 | -2.00 | -1.98 | -1.80 | -1.45 | -1.37 | -1.06 | -1.15 |
| Afu3g12180 | AFUA_3g12180 | C6 transcription factor, putative | -0.28 | -0.15 | -2.03 | -2.11 | -1.96 | -2.02 | -1.44 | -1.45 | -1.05 | -1.34 |
| Afu6g09740 | AFUA_6g09740 | thioredoxin reductase GliT | -0.26 | -0.11 | -2.03 | -1.77 | -2.22 | -1.98 | -1.75 | -1.82 | -0.90 | -1.39 |
| Afu5g11190 | AFUA_5g11190 | conserved hypothetical protein | -0.03 | 0.46 | -0.55 | -0.65 | -1.53 | -1.55 | -1.36 | -1.23 | -1.27 | -1.29 |
| Afu8g01340 | AFUA_8g01340 | MFS sugar transporter, putative | -0.06 | -0.01 | -1.54 | -1.74 | -1.35 | -1.59 | -1.38 | -1.27 | -1.26 | -1.38 |
| Afu8g05980 | AFUA_8g05980 | protein kinase, putative | NaN | -0.08 | -1.60 | -1.27 | -1.90 | -1.47 | -1.51 | -1.52 | -1.21 | -1.86 |
| Afu2g14430 | AFUA_2g14430 | cytochrome p450, putative | NaN | NaN | -1.41 | -1.43 | -1.89 | -1.69 | -1.44 | -1.45 | -1.36 | -1.59 |
| Afu5g01420 | AFUA_5g01420 | conserved hypothetical protein | 0.21 | 0.00 | -1.14 | -0.92 | -1.44 | -1.32 | -1.48 | -1.34 | -1.41 | -1.68 |
| Afu2g05990 | AFUA_2g05990 | conserved hypothetical protein | 0.35 | 0.54 | -1.01 | -1.13 | -1.34 | -1.54 | -1.42 | -1.36 | -1.17 | -1.40 |
| Afu5g06240 | AFUA_5g06240 | alcohol dehydrogenase, putative | -0.01 | 0.24 | -1.26 | -1.37 | -1.59 | -1.70 | -1.66 | -1.21 | -1.17 | -1.25 |
| Afu8g04860 | AFUA_8g04860 | GPI anchored glycoprotein, putative | -0.10 | NaN | -1.29 | -1.03 | -1.54 | -1.55 | -1.41 | -1.21 | -1.00 | -1.34 |
| Afu1g01490 | AFUA_1g01490 | NACHT domain protein | -0.03 | 0.42 | -1.23 | -1.13 | -1.34 | -1.25 | -1.45 | -1.21 | -0.96 | -1.20 |
| Afu2g05690 | AFUA_2g05690 | conserved hypothetical protein | NaN | NaN | -1.17 | -1.13 | -0.76 | -0.79 | -0.23 | -0.37 | 0.26 | 0.09 |
| Afu5g01990 | AFUA_5g01990 | BYS1 domain protein, putative | NaN | 0.15 | -1.54 | -1.19 | -1.20 | -0.98 | -0.02 | -0.13 | 0.07 | -0.12 |
| Afu6g00660 | AFUA_6g00660 | FAD binding domain protein | 0.03 | 0.00 | -0.63 | -0.55 | -0.84 | -0.78 | -0.24 | -0.23 | 0.41 | 0.60 |
| Afu3g01590 | AFUA_3g01590 | conserved hypothetical protein | 0.18 | -0.07 | -0.86 | -0.74 | -1.01 | -0.89 | -0.36 | -0.30 | 0.12 | 0.18 |
| Afu4g01290 | AFUA_4g01290 | endo-chitosanase, pseudogene | NaN | NaN | -1.03 | -0.87 | -1.05 | -0.81 | -0.14 | -0.01 | 0.32 | 0.53 |
| Afu3g14920 | AFUA_3g14920 | TAM domain methyltransferase, putative | NaN | NaN | -1.03 | -0.76 | -1.16 | -1.00 | -0.26 | -0.19 | 0.49 | 0.54 |
| Afu4g14250 | AFUA_4g14250 | hypothetical protein | NaN | NaN | -1.60 | -1.85 | -1.35 | -1.49 | -0.71 | -0.85 | -0.06 | 0.08 |
| Afu6g12450 | AFUA_6g12450 | chaperone/heat shock protein Awh11 | 1.07 | 0.18 | -1.90 | -1.34 | -1.36 | -1.35 | -1.18 | -1.22 | -0.90 | -1.24 |
| Afu2g00720 | AFUA_2g00720 | aldehyde dehydrogenase, putative | NaN | NaN | -1.96 | -1.55 | -1.88 | -1.67 | -1.07 | -1.11 | -0.78 | -0.89 |
| Afu8g05600 | AFUA_8g05600 | conserved hypothetical protein | 1.47 | 0.14 | -1.47 | -1.44 | -1.35 | -1.42 | -1.04 | -0.93 | -0.55 | -1.12 |
| Afu3g01260 | AFUA_3g01260 | acetyltransferase, GNAT family family | NaN | NaN | -1.37 | -1.24 | -0.81 | -0.88 | -0.33 | -0.44 | -0.29 | -0.24 |
| Afu3g09260 | AFUA_3g09260 | conserved hypothetical protein | NaN | 0.24 | -1.49 | -1.18 | -1.06 | -1.13 | -0.72 | -0.74 | -0.32 | -0.54 |
| Afu5g02320 | AFUA_5g02320 | conserved hypothetical protein | 0.72 | 0.19 | -0.93 | -0.89 | -1.12 | -1.01 | -0.61 | -0.50 | -0.42 | -0.38 |
| Afu4g12590 | AFUA_4g12590 | conserved hypothetical protein | 0.12 | -0.08 | -1.45 | -1.39 | -0.78 | -0.74 | -0.75 | -0.75 | -0.68 | -0.89 |
| Afu8g01710 | AFUA_8g01710 | antigenic thaumatin domain protein, putative | NaN | 0.16 | -1.80 | -1.51 | -0.80 | -0.60 | -0.68 | -0.56 | -0.45 | -0.55 |
| Afu5g02110 | AFUA_5g02110 | conserved hypothetical protein | 0.45 | -0.07 | -1.67 | -1.40 | -0.83 | -0.69 | -0.59 | -0.61 | -0.43 | -0.66 |
| Afu7g05350 | AFUA_7g05350 | delta-9 fatty acid desaturase; stearoyl-CoA desaturase | -0.20 | 0.13 | -1.48 | -1.28 | -1.59 | -1.30 | -1.03 | -0.76 | -0.60 | -0.51 |
| Afu4g13770 | AFUA_4g13770 | glycosyl hydrolase, putative | NaN | NaN | -1.48 | -0.99 | -1.47 | -1.49 | -0.97 | -0.92 | -0.53 | -0.72 |
| Afu3g12200 | AFUA_3g12200 | small oligopeptide transporter, OPT family | 0.14 | -0.11 | -1.25 | -1.25 | -1.40 | -1.32 | -1.05 | -0.73 | -0.62 | -0.71 |
| Afu6g13390 | AFUA_6g13390 | FAD dependent oxidoreductase, putative | NaN | NaN | -1.71 | -1.30 | -1.77 | -1.25 | -1.06 | -0.94 | -0.80 | -0.95 |
| Afu6g02220 | AFUA_6g02220 | MFS toxin efflux pump, putative | 0.69 | -0.01 | -1.41 | -1.18 | -1.56 | -1.28 | -0.90 | -1.00 | -0.99 | -1.07 |
| Afu6g02180 | AFUA_6g02180 | GNAT family acetyltransferase, putative | 0.05 | 0.24 | -1.53 | -1.31 | -1.41 | -1.18 | -0.89 | -0.82 | -1.00 | -0.96 |
| Afu4g01140 | AFUA_4g01140 | MFS multidrug transporter, putative | 0.10 | -0.23 | -1.32 | -1.56 | -1.47 | -1.41 | -1.21 | -1.36 | -1.02 | -1.34 |
| Afu3g10760 | AFUA_3g10760 | phosphoketolase, putative | 0.42 | -0.24 | -1.47 | -1.50 | -1.38 | -1.42 | -1.03 | -1.13 | -0.97 | -1.16 |
| Afu6g03890 | AFUA_6g03890 | spore-specific catalase CatA | 0.00 | 0.01 | -1.57 | -1.34 | -1.30 | -1.32 | -1.18 | -1.17 | -1.13 | -1.11 |
| Afu2g08290 | AFUA_2g08290 | conserved hypothetical protein | -0.04 | -0.08 | -1.26 | -1.17 | -1.37 | -1.14 | -1.21 | -1.20 | -1.06 | -1.22 |
| Afu7g05060 | AFUA_7g05060 | MgtC/SapB family membrane protein | 0.18 | 0.06 | -1.26 | -1.10 | -1.48 | -1.36 | -1.29 | -1.06 | -0.87 | -1.18 |
| Afu5g09970 | AFUA_5g09970 | 67 kDa myosin-cross-reactive antigen family protein | 0.23 | 0.11 | -1.20 | -1.12 | -1.49 | -1.25 | -1.19 | -1.04 | -0.90 | -1.04 |
| Afu3g03940 | AFUA_3g03940 | 2,3-diketo-5-methylthio-1-phosphopentane phosphatase, putative | 0.08 | -0.02 | -1.39 | -1.11 | -1.29 | -1.33 | -1.19 | -1.08 | -0.89 | -0.96 |
| Afu3g00500 | AFUA_3g00500 | integral membrane protein | 0.61 | 0.43 | -1.13 | -0.68 | -1.26 | -1.03 | -1.23 | -0.79 | -0.77 | -0.76 |
| Afu5g08190 | AFUA_5g08190 | conserved hypothetical protein | NaN | 0.01 | -1.02 | -1.29 | -1.10 | -1.14 | -1.02 | -1.01 | -0.94 | -0.74 |
| Afu5g04250 | AFUA_5g04250 | homocysteine synthase CysD | 0.35 | 0.12 | -1.18 | -1.36 | -1.10 | -1.07 | -0.92 | -0.90 | -0.71 | -0.62 |
| Afu8g04550 | AFUA_8g04550 | sulfonate biosynthesis enzyme, putative | 0.81 | 0.09 | -1.42 | -1.36 | -1.06 | -1.24 | -0.92 | -0.79 | -0.87 | -0.96 |
| Afu1g09750 | AFUA_1g09750 | aldehyde reductase (AKR1), putative | 0.69 | 0.15 | -1.51 | -1.22 | -0.97 | -1.00 | -0.83 | -0.77 | -0.67 | -0.90 |
| Afu6g12460 | AFUA_6g12460 | conserved hypothetical protein | 0.93 | 0.18 | -1.24 | -1.20 | -1.27 | -1.22 | -1.14 | -1.08 | -0.97 | -0.96 |
| Afu5g09960 | AFUA_5g09960 | GPI anchored protein, putative | 0.49 | 0.08 | -1.17 | -1.10 | -1.43 | -1.18 | -0.90 | -0.85 | -0.55 | -0.99 |
| Afu4g07860 | AFUA_4g07860 | hypothetical protein | 0.52 | 0.17 | -1.23 | -0.93 | -1.36 | -1.22 | -1.15 | -1.00 | -0.61 | -1.17 |
| Afu8g01290 | AFUA_8g01290 | hypothetical protein | 0.00 | 0.15 | -1.08 | -1.13 | -1.21 | -1.36 | -0.74 | -0.82 | -0.66 | -0.98 |
| Afu4g08960 | AFUA_4g08960 | GPI anchored cell wall protein, putative | 0.19 | 0.10 | -0.91 | -0.73 | -1.09 | -1.11 | -1.05 | -1.11 | -0.72 | -0.89 |
| Afu2g01940 | AFUA_2g01940 | conserved hypothetical protein | 0.11 | 0.28 | -0.94 | -0.86 | -1.30 | -1.08 | -0.87 | -0.89 | -0.61 | -0.88 |
| Afu6g10220 | AFUA_6g10220 | small oligopeptide transporter, OPT family | 0.23 | 0.18 | -1.14 | -1.04 | -1.41 | -1.19 | -1.02 | -0.88 | -0.82 | -0.84 |
| Afu7g05070 | AFUA_7g05070 | FAD dependent oxidoreductase, putative | 0.10 | 0.21 | -1.07 | -0.92 | -1.28 | -1.02 | -1.04 | -0.85 | -0.77 | -0.80 |
| Afu8g02650 | AFUA_8g02650 | ABC multidrug transporter, putative | 0.09 | 0.13 | -0.74 | -0.96 | -1.12 | -1.19 | -1.21 | -1.05 | -0.98 | -1.13 |
| Afu2g08280 | AFUA_2g08280 | NADP-dependent malic enzyme MaeA | 0.23 | 0.06 | -1.10 | -1.08 | -1.06 | -0.97 | -1.04 | -0.85 | -1.02 | -1.00 |
| Afu5g06230 | AFUA_5g06230 | GABA permease, putative | 0.25 | -0.05 | -0.94 | -1.03 | -1.37 | -1.30 | -1.05 | -0.74 | -0.97 | -1.07 |
| Afu4g01560 | AFUA_4g01560 | MFS myo-inositol transporter, putative | 0.32 | -0.04 | 0.03 | -0.11 | -0.71 | -1.04 | -1.13 | -1.13 | -1.13 | -1.14 |
| Afu4g08950 | AFUA_4g08950 |  | 0.58 | 0.32 | -0.04 | 0.31 | -0.63 | -0.61 | -0.80 | -0.73 | -0.61 | -0.77 |
| Afu4g14640 | AFUA_4g14640 | low affinity iron transporter, putative | 0.88 | 0.12 | 0.18 | 0.14 | -0.68 | -0.76 | -0.91 | -0.95 | -0.84 | -1.27 |
| Afu6g10410 | AFUA_6g10410 | vacuolar protein sorting protein (VPS11), putative | 0.63 | 0.48 | -0.49 | -0.52 | -0.38 | -0.50 | -0.50 | -0.51 | -0.59 | -0.78 |
| Afu1g09040 | AFUA_1g09040 | intermediate filament protein (Mdm1), putative | 0.21 | 0.49 | -0.74 | -0.70 | -0.91 | -0.81 | -0.71 | -0.77 | -0.81 | -0.86 |
| Afu1g14880 | AFUA_1g14880 | N-acylethanolamine amidohydrolase, putative | 0.35 | 0.22 | -0.75 | -0.61 | -0.84 | -0.68 | -0.83 | -0.86 | -1.00 | -1.19 |
| Afu1g12320 | AFUA_1g12320 | hypothetical protein | 0.59 | 0.39 | -0.52 | -0.29 | -0.79 | -0.66 | -0.68 | -0.74 | -0.78 | -1.01 |
