# Supplementary material for: The Temporal Dynamics of Differential Gene Expression in Aspergillus fumigatus Interacting with Human Immature Dendritic Cells In Vitro
Source: PLoS One. 2011 Jan 14;6(1):e16016. doi: 10.1371/journal.pone.0016016 (PMC3021540; doi:10.1371/journal.pone.0016016)
Supplement: Table S2 — Gene Ontology analysis of biological processes up-regulated by A. fumigatus during infection of iDC1. (DOC) [file pone.0016016.s003.doc]

**Table S2 - Gene Ontology analysis of biological processes up-regulated by *A. fumigatus* during infection of iDC^1^**

| **L-phenylalanine catabolism (p: 0.003)** | | **Hypusine biosynthesis from peptidyl-lysine (p: 0.017)** | |
| --- | --- | --- | --- |
| ^a^Freq. ^b^Array 2 (2.6) ^c^Genome 5 (0.1) | | ^a^Freq. ^b^Array 1 (1.3) ^c^Genome 1 (0.02) | |
| **Gene ID** | **Gene Annotation** | **Gene ID** | **Gene Annotation** |
| Afu2g04230 | fumarylacetoacetate hydrolase FahA | Afu5g01740 | deoxyhypusine synthase |
| Afu2g04200 | 4-hydroxyphenylpyruvate dioxygenase | **Cytosine transport (p: 0.017)** | |
|  |  | ^a^Freq. Array 1 (1.3) Genome 1 (0.02) | |
| **Tyrosine catabolism (p: 0.003)** | | Afu2g09860 | purine-cytosine permease |
| ^a^Freq. Array 2 (2.6) Genome 5 (0.1) | |  |  |
| Afu2g04230 | fumarylacetoacetate hydrolase FahA | **Mannose inositol phosphoceramide metabolism (p: 0.017)** | |
|  |  | ^a^Freq. Array 1 (1.3) Genome 1 (0.02) | |
| Afu2g04200 | 4-hydroxyphenylpyruvate dioxygenase | Afu6g04680 | MIPC synthase subunit (SurA) |
| **Peptide transport (p: 0.004)** | | **Para-aminobenzoic acid metabolism (p: 0.017)** | |
| ^a^Freq. Array 2 (2.6) Genome 6 (0.1) | | ^a^Freq. Array 1 (1.3) Genome 1 (0.02) | |
| Afu7g01490 | MFS peptide transporter Ptr2 | Afu6g04820 | para-aminobenzoate synthase PabaA |
| Afu4g00830 | MFS peptide transporter | **Purine transport (p: 0.017)** | |
|  |  | ^a^Freq. Array 1 (1.3) Genome 1 (0.02) | |
| **Pathogenesis (p: 0.007)** | | Afu2g09860 | purine-cytosine permease |
| ^a^Freq. Array 4 (5.3) Genome 44 (0.1) | |  |  |
| Afu2g03830 | allergen Asp F4 | **Ribosome biogenesis (p: 0.031)** | |
|  |  | ^a^Freq. Array 2 (2.6) Genome 16 (0.4) | |
| Afu6g04820 | para-aminobenzoate synthase PabaA | Afu8g05430 | ribosome biogenesis protein Noc4 |
| Afu5g02330 | major allergen and cytotoxin AspF1 | Afu2g08480 | ATP-dependent RNA helicase Mrh4 |
| Afu2g04200 | 4-hydroxyphenylpyruvate dioxygenase | **Peptidyl-arginine modification (p: 0.035)** | |
|  |  | ^a^Freq. Array 1 (1.3) Genome 2 (0.05) | |
| **Drug Transport (p: 0.009)** | | Afu1g06190 | histone H4 arginine methyltransferase RmtA |
| ^a^Freq. Array 3 (4.0) Genome 25 (0.6) | |  |  |
| Afu1g13800 | MFS multidrug transporter | **rRNA processing (p: 0.035)** | |
|  |  | ^a^Freq. Array 3 (4.0) Genome 41 (1.0) | |
| Afu2g16860 | MFS multidrug transporter | Afu1g02610 | rRNA processing protein |
| Afu7g00390 | MFS multidrug transporter | Afu8g04820 | ribonuclease P complex subunit Pop2 |
| **Arginine catabolism to ornithine (p: 0.017)** | | Afu2g05560 | exonuclease, putative |
| ^a^Freq. Array 1 (1.3) Genome 1 (0.02) | |  |  |
| Afu3g11430 | Arginase | **Glucan metabolism (p: 0.048)** | |
|  |  | ^a^Freq. Array 2 (2.6) Genome 20 (0.5) | |
| **Folic acid biosynthesis(p: 0.018)** | | Afu8g05610 | cell wall glucanase (Scw11) |
| ^a^Freq. Array 1 (1.3) Genome 1 (0.02) | |  |  |
| Afu6g04820 | para-aminobenzoate synthase PabaA | Afu6g08510 | cell wall glucanase |

^1^ GO Terms with P values <0.05 are shown

^a^ Frequency of genes (number of genes (%of total)) in the array data or the total genome

^b^ There were 75 up-regulated genes used by MEV for the GO analysis

^c^ There are 4219 annotated genes in the *A. fumigatus* genome
