# Supplementary material for: The Temporal Dynamics of Differential Gene Expression in Aspergillus fumigatus Interacting with Human Immature Dendritic Cells In Vitro
Source: PLoS One. 2011 Jan 14;6(1):e16016. doi: 10.1371/journal.pone.0016016 (PMC3021540; doi:10.1371/journal.pone.0016016)
Supplement: Table S3 — Gene Ontology analysis of biological processes down-regulated by A. fumigatus during infection of iDC1. (DOC) [file pone.0016016.s004.doc]

**Table S3 -** Gene Ontology analysis of biological processes down-regulated by *A. fumigatus* during infection of iDC^1^

| **Fermentation (p: 0.002)** | | **Carbohydrate metabolism (p: 0.021)** | |
| --- | --- | --- | --- |
| ^a^Freq. ^b^Array 2 (7.4) ^c^Genome 11 (0.3) | | ^a^Freq. ^b^Array 2 (7.4) ^c^Genome 36 (0.9) | |
| **Gene ID** | **Gene Annotation** | **Gene ID** | **Gene Annotation** |
| Afu5g06240 | alcohol dehydrogenase | Afu4g13770 | glycosyl hydrolase |
| Afu2g00720 | Aldehyde dehydrogenase | Afu3g10760 | phosphoketolase |
| **Sulfur metabolism (p: 0.0057)** | | **Cell adhesion (p: 0.025)** | |
| ^a^Freq. Array 2 (7.4) Genome 18 (0.4) | | ^a^Freq. Array 1 (3.7) Genome 4 (0.1) | |
| Afu6g10220 | small oligopeptide transporter, OPT family | Afu6g12450 | chaperone/heat shock protein Hsp12 |
| Afu3g12200 | small oligopeptide transporter, OPT family | **Amino acid metabolism (p: 0.025)** | |
|  |  | ^a^Freq. Array 1 (3.7) Genome 4 (0.1) | |
| **Transport (p: 0.016)** | | Afu2g08280 | NADP-dependent malic enzyme MaeA |
| ^a^Freq. Array 4 (14.8) Genome 157 (3.7) | |  |  |
| Afu6g02220 | MFS toxin efflux pump | **Nuclear migration (p: 0.025)** | |
|  |  | ^a^Freq. Array 1 (3.7) Genome 4 (0.1) | |
| Afu5g06230 | GABA permease | Afu1g09040 | intermediate filament protein (Mdm1) |
| Afu8g02650 | ABC multidrug transporter | **Myo-inositol transport (p: 0.025)** | |
|  |  | ^a^Freq. Array 1 (3.7) Genome 5 (0.1) | |
| Afu6g09710 | MFS gliotoxin efflux transporter GliA | Afu4g01560 | MFS myo-inositol transporter |
| **Hyperosmotic response (p: 0.019)** | | **Golgi to endosome transport (p: 0.025)** | |
| ^a^Freq. Array 1 (3.7) Genome 3 (0.1) | | ^a^Freq. Array 1 (3.7) Genome 5 (0.1) | |
| Afu6g12450 | chaperone/heat shock protein Hsp12 | Afu6g10410 | vacuolar protein sorting protein (VPS11) |
| **Response to desiccation (p: 0.019)** | | **Urea metabolism (p: 0.025)** | |
| ^a^Freq. Array 1 (3.7) Genome 3 (0.1) | | ^a^Freq. Array 1 (3.7) Genome 5 (0.1) | |
| Afu6g12450 | chaperone/heat shock protein Hsp12 | Afu1g14880 | N-acylethanolamine amidohydrolase |
| **Response to oxidative stress (p: 0.019)** | | **Homotypic vacuole fusion, non-autophagic (p: 0.04)** | |
| ^a^Freq. Array 2 (7.4) Genome 34 (0.8) | | ^a^Freq. Array 1 (3.7) Genome 7 (0.2) | |
| Afu6g03890 | spore-specific catalase CatA | Afu6g10410 | vacuolar protein sorting protein (VPS11) |
| Afu6g12450 | chaperone/heat shock protein Hsp12 |  |  |

^1^ GO Terms with P values <0.05 are shown

^a^ Frequency of genes (number of genes (%)) in the array data or the total genome

^b^ There were 27 down-regulated genes used by MEV for the GO analysis

^c^ There are 4219 annotated genes in the *A. fumigatus* genome
