# Supplementary material for: The Temporal Dynamics of Differential Gene Expression in Aspergillus fumigatus Interacting with Human Immature Dendritic Cells In Vitro
Source: PLoS One. 2011 Jan 14;6(1):e16016. doi: 10.1371/journal.pone.0016016 (PMC3021540; doi:10.1371/journal.pone.0016016)
Supplement: Table S4 — Differentially expressed A. fumigatus genes located in the subtelomeric regions of each chromosome during infection of iDC. (DOC) [file pone.0016016.s005.doc]

**Table S4 Differentially expressed *A. fumigatus* genes located in the subtelomeric regions of each chromosome during infection of iDC**

| **ORF** | **Gene Name** | **^1^Infection Cluster** | **Expression Data (Log2)** | | | |
| --- | --- | --- | --- | --- | --- | --- |
|  |  |  | 3h | 6h | 9h | 12 h |
| **Chromosome I** | | | | | | |
| AFUA_2g03860 | plasma membrane low affinity zinc ion transporter, putative |  | -1.18 | -1.29 | -1.33 | -1.08 |
| AFUA_4g09580 | major allergen Asp F2 |  | 1.25 | 1.42 | 1.20 | 1.34 |
| AFUA_1g17060 | HLH DNA binding domain protein, putative |  | 1.84 | 1.20 | 0.56 | 0.12 |
| **Chromosome II** | | | | | | |
| AFUA_2g00720 | aldehyde dehydrogenase, putative |  | -1.76 | -1.78 | -1.09 | -0.84 |
| AFUA_2g16750 | nonsense-mediated mRNA decay protein 3 |  | 1.24 | 1.45 | 1.01 | 0.85 |
| AFUA_2g16860 | MFS multidrug transporter, putative |  | 1.49 | 2.10 | 2.39 | 1.77 |
| **Chromosome III** | | | | | | |
| AFUA_3g00500 | integral membrane protein | 31 | -0.91 | -1.15 | -1.01 | -0.77 |
| AFUA_3g01030 | RTA1 domain protein, putative | 31 | 1.80 | 1.37 | 0.98 | 0.82 |
| AFUA_3g14920 | TAM domain methyltransferase, putative |  | -0.90 | -1.08 | -0.23 | 0.52 |
| **Chromosome IV** | | | | | | |
| AFUA_4g00610 | aryl-alcohol dehydrogenase, putative |  | -0.01 | -0.29 | 0.33 | 1.27 |
| AFUA_4g00750 | conserved hypothetical protein |  | -0.22 | 1.12 | 1.77 | 2.68 |
| AFUA_4g00830 | MFS peptide transporter, putative |  | -0.05 | 0.51 | 1.19 | 1.27 |
| AFUA_4g14250 | hypothetical protein |  | -1.73 | -1.42 | -0.78 | 0.01 |
| AFUA_4g14640 | low affinity iron transporter, putative |  | 0.16 | -0.72 | -0.93 | -1.06 |
| **Chromosome V** | | | | | | |
| AFUA_5g00720 | GNAT family acetyltransferase, putative | 46 | -0.41 | -0.24 | 0.34 | 1.32 |
| AFUA_5g01420 | conserved hypothetical protein | 47 | -1.03 | -1.38 | -1.41 | -1.54 |
| AFUA_5g14430 | Unknown Function |  | 0.77 | 1.20 | 1.84 | 1.65 |
| AFUA_5g14660 | GABA permease, putative | 53 | 0.11 | -0.35 | 0.49 | 0.96 |
| AFUA_5g15010 | arsenite efflux transporter (ArsB), putative |  | 1.28 | 1.02 | 0.81 | 0.44 |
| **Chromosome VI** | | | | | | |
| AFUA_6g00430 | IgE-binding protein |  | -0.65 | 1.16 | 2.34 | 2.11 |
| AFUA_6g00620 | GPI anchored hypothetical protein | 55 | -0.69 | -0.20 | 0.38 | 0.91 |
| AFUA_6g00630 | MFS transporter, putative | 55 | -0.42 | -0.54 | 0.76 | 1.31 |
| AFUA_6g00640 | integral membrane protein | 55 | -0.30 | -0.48 | 0.54 | 1.01 |
| AFUA_6g00660 | FAD binding domain protein |  | -0.59 | -0.81 | -0.24 | 0.51 |
| AFUA_6g13390 | FAD dependent oxidoreductase, putative |  | -1.51 | -1.51 | -1.00 | -0.88 |
| AFUA_6g14260 | U3 small nucleolar ribonucleoprotein protein Lcp5, putative |  | 1.26 | 1.53 | 1.24 | 1.01 |
| AFUA_6g14510 | monooxygenase, putative |  | 2.15 | 2.15 | 1.44 | 1.13 |
| **Chromosome VII** | | | | | | |
| AFUA_7g00120 | metallo-beta-lactamase domain protein | 63 | 0.14 | 0.43 | 1.42 | 1.53 |
| AFUA_7g00170 | dimethylallyl tryptophan synthase GliD1 | 63 | -0.46 | 0.62 | 1.87 | 1.77 |
| AFUA_7g00200 | conserved hypothetical protein | 63 | -0.34 | 0.19 | 1.16 | 1.11 |
| AFUA_7g00340 | metallo-beta-lactamase domain protein, putative |  | 2.10 | 2.67 | 2.92 | 2.85 |
| AFUA_7g00390 | MFS multidrug transporter, putative |  | 0.25 | 0.70 | 0.89 | 0.91 |
| AFUA_7g00580 | conserved hypothetical protein |  | -0.29 | -0.20 | 0.59 | 0.71 |
| AFUA_7g00930 | integral membrane protein |  | 1.11 | 0.93 | 0.64 | 0.35 |
| AFUA_7g00990 | transcriptional activator of ethanol catabolism AlcS |  | 0.37 | 1.77 | 2.28 | 1.48 |
| AFUA_7g01000 | potassium-activated aldehyde dehydrogenase Ald4, putative |  | 0.61 | 2.84 | 3.79 | 3.41 |
| **Chromosome VIII** | | | | | | |
| AFUA_8g00610 | cell surface protein Mas1, putative | 72 | -0.26 | 2.33 | 3.57 | 2.83 |
| AFUA_8g00720 | amino acid transporter, putative |  | 1.10 | 1.43 | 1.18 | 0.97 |
| AFUA_8g01030 | conserved hypothetical protein | 73 | 0.23 | 0.94 | 1.30 | 1.74 |
| AFUA_8g01260 | hypothetical protein |  | 0.96 | 1.05 | 0.88 | 0.62 |

^1^Infection clusters identified by Mc Donagh *et al*. [11] using SMURF (<http://jcvi.org/smurf/index.php>)
