# Supplementary material for: The Temporal Dynamics of Differential Gene Expression in Aspergillus fumigatus Interacting with Human Immature Dendritic Cells In Vitro
Source: PLoS One. 2011 Jan 14;6(1):e16016. doi: 10.1371/journal.pone.0016016 (PMC3021540; doi:10.1371/journal.pone.0016016)
Supplement: Table S5 — Oligonucleotides used for quantitative real time PCR analysis of A. fumigatus gene expression. (DOC) [file pone.0016016.s006.doc]

**Table S5 Oligonucleotides used for quantitative real time PCR analysis of *A. fumigatus* gene expression**

| **Locus ID/Gene** | **Forward Primer** | **Reverse Primer** |
| --- | --- | --- |
| Afua_6g04740/ Act A | GTCAACCGTTGCCCTTG | TCGTCAAGTTAGCGTTTCATTGT |
| Afu4g09580/ Asp F2 | TCTGTGATCGCAGCTACACC | ACAGCAGGCACATGGTACAG |
| Afu2g03860/ putative | GTGGAGTCCAAAAGCCACAT | AGGCCAATGAAGATCGAATG |
| Afu7g00340/ putative | TCGACCGAATTCTTGAAACC | CCCAAACAACTTCTGCACCT |
| Afu3g10680/putative | AATGTCTGCCGAGGAGAAGA | ATCTGACCGCATGAGAAACC |
| Afu7g06540/ putative | TCTGTTGCGGTCAAATACCA | GCTTCATAGGGCCGTAAACA |
| Afu6g09730/ GliF | GTCTAAAGGCGCTGGACTTG | CGGTGTCGATCTCAGAGACA |
| Afu3g12180/ putative | GGATGCGGAGGAGAATGATA | CTGGAGGTTTCGAAGAGTGC |
| Afu6g09710/ GliA | TTTGCGATCAACGAACTCTG | CCCTTGACGGACTGGAAGTA |
| Afu2g11120/ putative | CTTCCAGAGCTTTCCAGACG | CTGTGTTGGAAACGGGAGAT |
| Afu1g06390/ EF-1 | AGGTCATCGTCCTCAACCAC | ACCGGACTTGATGAACTTGG |
| Afu5g01970/ Gpd A | AACATCATTCCCAGCTCGAC | ACGTTGGAGGTAGGAACACG |
